# A mixed‐method systematic review and meta‐analysis of the influences of food environments and food insecurity on obesity in high‐income countries

**DOI:** 10.1002/fsn3.2969

**Published:** 2022-08-05

**Authors:** Fatemeh Eskandari, Amelia A. Lake, Kelly Rose, Mark Butler, Claire O'Malley

**Affiliations:** ^1^ Centre for Public Health Research, School of Health and Life Sciences Teesside University Middlesbrough UK; ^2^ Fuse ‐ The Centre for Translational Research in Public Health Newcastle upon Tyne UK

**Keywords:** food environments, food insecurity, obesity, overweight

## Abstract

Obesity remains a serious public health concern in rich countries and the current obesogenic food environments and food insecurity are predictors of this disease. The impact of these variables on rising obesity trends is, however, mixed and inconsistent, due to measurement issues and cross‐sectional study designs. To further the work in this area, this review aimed to summarize quantitative and qualitative data on the relationship between these variables, among adults and children across high‐income countries. A mixed‐method systematic review was conducted using 13 electronic databases, up to August 2021. Two authors independently extracted data and evaluated quality of publications. Random‐effects meta‐analysis was used to estimate the odds ratio (OR) for the association between food insecurity and obesity. Where statistical pooling for extracted statistics related to food environments was not possible due to heterogeneity, a narrative synthesis was performed. Meta‐analysis of 36,113 adults and children showed statistically significant associations between food insecurity and obesity (OR: 1.503, 95% confidence interval: 1.432–1.577, *p* < .05). Narrative synthesis showed association between different types of food environments and obesity. Findings from qualitative studies regarding a reliance on energy‐dense, nutrient‐poor foods owing to their affordability and accessibility aligned with findings from quantitative studies. Results from both qualitative and quantitative studies regarding the potential links between increased body weight and participation in food assistance programs such as food banks were supportive of weight gain. To address obesity among individuals experiencing food insecurity, wide‐reaching approaches are required, especially among those surrounded by unhealthy food environments which could potentially influence food choice.

## Graphical abstract

This review aimed to summarize quantitative and qualitative data on the relationship betweenobesogenic food environments, food insecurity, and obesity among adults and children across high‐income countries. A mixed‐method systematic review was conducted using 13 electronic databases, up to August 2021.

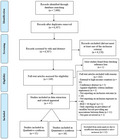

## 1 INTRODUCTION

Obesity remains a serious public health concern in high‐income countries due to its alarming prevalence and costly long‐term health problems (Lee et al., 2021). Overweight and obesity are defined by World Health Organization (WHO) as “abnormal or excessive fat accumulation that presents a risk to health” (Ryan et al., 2020). The prevalence of overweight and obesity has dramatically increased since the 1980s (Swinburn et al., 2019). While hunger is increasing in many countries, it is estimated that 2 billion people are affected by either overweight or obesity (Ryan et al., 2020). Overweight or obesity is one of the major risk factors for noncommunicable diseases (NCDs) worldwide, including cardiovascular diseases, diabetes, and cancers (Kluge et al., 2020). In the light of COVID‐19 (the coronavirus disease 2019) pandemic, increasing recent studies have also linked obesity with a higher risk of hospitalization, severe symptoms, and death from this disease (Department of Health and Social Care, 2020; Sattar et al., 2020). The severity of these risks increase as body mass index (BMI) increases (Department of Health and Social Care, 2020). In more wealthy, developed countries, many efforts have been made thus far to tackle obesity, including the implementation of relevant national and international policies and programs. However, these measures have proved inadequate for controlling the obesity epidemic in these countries (Lee et al., 2021). Because of this rapid increase in obesity prevalence, it has been argued that the cause is most likely to be related to environmental changes rather than biological changes (Jeffery et al., 2006; Leal & Chaix, 2011). Modifying the food environments has been recognized to have a vital role in shaping individuals' eating behaviors and purchasing (Wang et al., 2019). Food environments are defined as “the availability, affordability, convenience, and desirability of various foods surrounding individuals” (Wang et al., 2019). A recent conceptual framework has been developed which defines personal and external food environment domains (Turner et al., 2020). Dimensions related to individuals, such as food accessibility, affordability, convenience, and desirability form the personal domain, whereas exogenous dimensions such as food availability, prices, vendor, and product properties make the external domain (Turner et al., 2020).

Food insecurity is also thought to play a role in the rising trend of obesity and related NCDs (Keenan et al., 2020; Kirkman et al., 2020). Food insecurity has been defined as “limited or uncertain availability of nutritionally adequate and safe foods or limited or uncertain ability to acquire acceptable foods in socially acceptable ways” (Taylor & Loopstra, 2016). The United Nations (UN) estimated that 135 million people around the world experienced severe food insecurity at the beginning of 2020 (before the coronavirus pandemic) (Covid‐19 and food security, 2020). This figure was estimated to be doubled to 265 million by the end of 2020 as a result of recent COVID‐19 crisis (Covid‐19 and food security, 2020) that has made clear disparities in the food supply and distribution chain, having a detrimental effect on availability and access (Power et al., 2020). Food insecurity contributes to both malnutrition and the paradox of obesity in high‐income countries (Penne & Goedemé, 2020). Evidence indicates that those from lower socioeconomic status groups can only afford energy‐dense food that is low in nutrients, causing obesity, impaired liver function, hypertension, and iron deficiency (Thompson et al., 2018). Significant disparities exist in access to healthier food as more socially deprived areas have more clusters of unhealthy food outlets (Kirkman et al., 2020). In neighborhoods with high prevalence of food insecurity, it is thought that the higher availability of cheap, high‐energy‐dense foods plays an important role in the relationship between weight status and food insecurity (Keenan et al., 2020). Due to high association between food insecurity and poverty, individuals with food insecurity are expected to belong to high‐poverty neighborhoods that have constrained access to healthy and nutritious foods (Ro & Osborn, 2018). There is a global “cost of living crisis” which is impacting households and their ability to purchase food (Hourston, 2022). Rising trends in food insecurity has led to the provision and inclusion of donated and surplus food by charity and third‐sector organizations, into the diets of people with low‐income conditions (Thompson et al., 2019). As the provision of food aid is growing and diversifying in high‐income countries, these not‐for‐profit retail outlets have been proposed to be incorporated into concepts of the food environments (Thompson et al., 2019). Participation in food assistance programs has been found to be associated with obesity (Ro & Osborn, 2018). A recent systematic review has indicated that the nutrition quality of food parcels is inconsistent, and is often poor compared with national nutritional recommendations (Oldroyd et al., 2022).

Available literature demonstrates that the association between food insecurity, food environments, and risk for overweight and obesity is ambiguous and inconsistent (Biadgilign et al., 2021; Chen et al., 2016; Morales & Berkowitz, 2016; Nettle & Bateson, 2019). Therefore, a comprehensive mixed‐methods systematic review and meta‐analysis was conducted to assess these relationships among adults and children across high‐income countries. The specific objectives of this review were to (a) explore how the food environment and food insecurity are associated with obesity among adults and children, (b) examine the role of food and nutrition assistance programs (as an additional contemporary aspect of the food environments) on the relationship between food insecurity and weight status, and (c) understand the gaps and limitations in the literature. This review was limited to high‐income countries as most data collection assessments to evaluate food environments have been conducted and validated in high‐income countries context over the past two decades. Furthermore, food environments in high‐income countries dramatically differ from those found in low‐ and middle‐income countries (LMICs). For example, rural households in LMICs usually obtain foods from informal market food environments which have limited schedules and highly seasonal food offerings. In contrast, consumers in high‐income countries mainly access formal market food environments, such as supermarkets, restaurants, and fast‐food chains (Ahmed et al., 2021).

To the best of authors' knowledge, this is the first mixed‐method systematic review that aimed to further the work in this area using both quantitative and qualitative studies. Mixed‐methods systematic reviews have become increasingly important as they provide a more complete basis for complex decision‐making than that currently offered by single‐method reviews to answer complex applied health and public health questions (Stern, Lizarondo, Carrier, Godfrey, et al., 2020). Therefore, the current mixed‐method review substantially differs from two recent systematic reviews and meta‐analyses which only included quantitative study designs to investigate links between food insecurity and weight status regardless of considering the impact of food environments and qualitative study designs in their studies that were not limited to high‐income countries (Moradi et al., 2019; Pourmotabbed et al., 2020). Building on these, we reviewed literature for both quantitative and qualitative studies restricted to high‐income countries up to August 2021, with a strong focus on considering the impact of both different food environments (objective or perceived measures) and food insecurity status on obesity.

## 2 METHODS

This mixed‐methods systematic review and meta‐analysis followed the Joanna Briggs Institute (JBI) Reviewers Manual 2020 (Aromataris & Munn, 2020) and the Preferred Reporting Items for Systematic reviews and Meta‐Analyses (PRISMA) statement (Moher et al., 2009)/Objectives, eligibility criteria, and methods of analysis were specified in advance and published in a *priory* protocol (PROSPERO [CRD42019124339]) (Eskandari et al., 2019).

### 2.1 Search strategy and selection of studies

**FIGURE 1:**
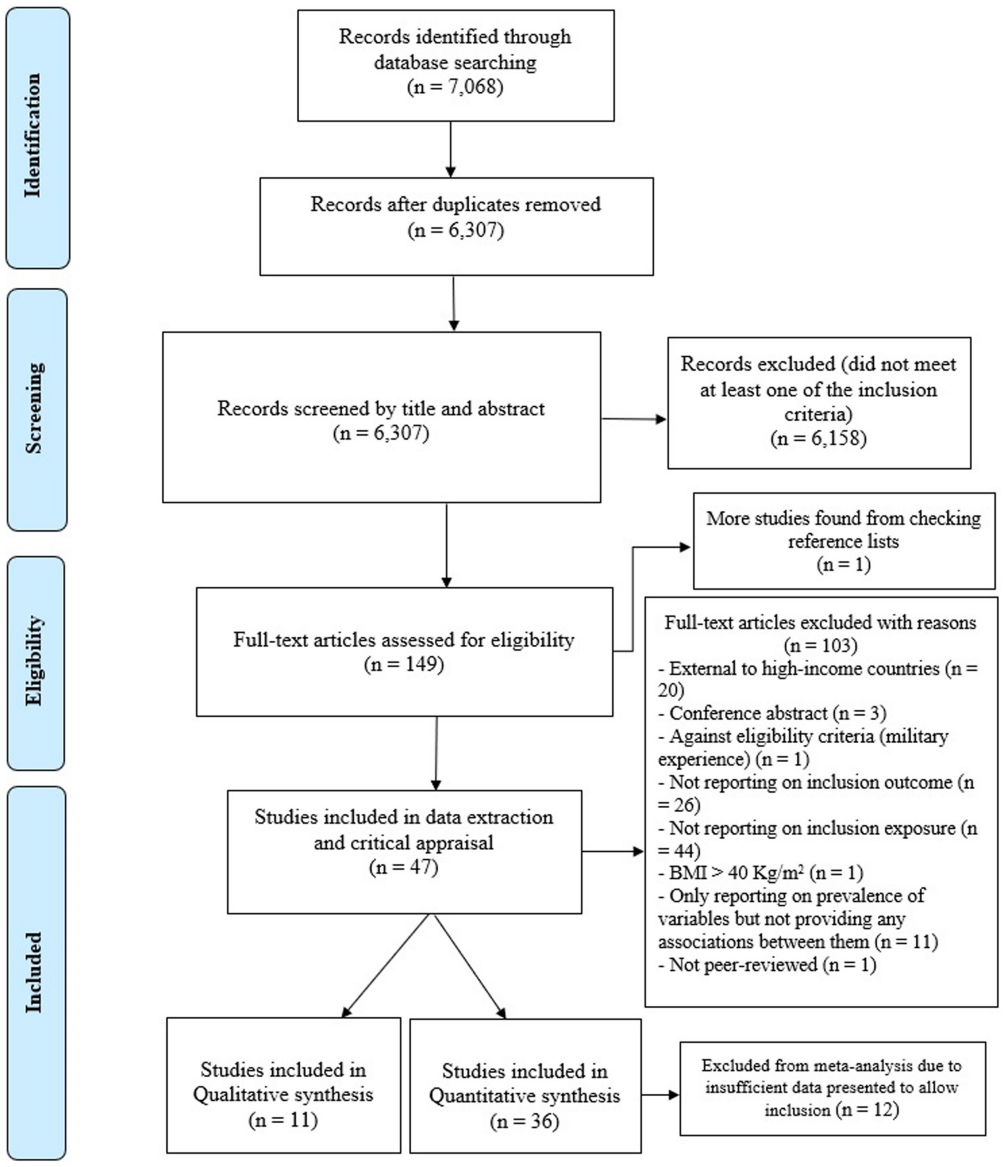
PRISMA flow diagram of the search and screening process for the current mixed‐method systematic review.

Thirteen electronic databases were searched including PubMed, CINAHL, EMBASE, MEDLINE, PsycINFO, ERIC, Scopus, Web of Science, EThoS, Cochrane Library, JBI Library, PROSPERO, and Google Scholar. Search strategy and full search example can be found in Tables S1 and S2. The reference lists of selected articles for critical appraisal were also checked for additional relevant studies. The searches were undertaken from January 2019 to 31 August 2021. The first author (FE) performed the searches and imported citations into Endnote version x9 for the screening process and for removing duplicates. The titles and abstracts were assessed separately by two investigators; all titles and abstracts were screened by the first author (FE) in the first screening stage. Twenty percent of titles and abstracts were then independently double screened by a second reviewer (KR). A third reviewer (CO) resolved any discrepancies by consensus or provided clarification. In the next stage, full‐text articles were evaluated against the eligibility and study quality criteria. The screening for full‐text papers was performed by (FE and KR). The PRISMA flow diagram shows the number of articles at each stage (Figure 1).

### 2.2 Eligibility criteria

Eligible study designs included observational studies such as cross‐sectional studies, cohort studies, and case–control studies, aimed at exploring the association between the food environment, food insecurity, and obesity in adults or children conducted in high‐income countries (as defined by the World Bank; The World Bank Group, 2021) published from 1992 onwards (as major studies related to the food environment and food insecurity began from this date). Furthermore, qualitative studies that investigated the perceptions and experiences of obesity arisen from food poverty/insecurity and unhealthy food environments were included. Systematic reviews were excluded, and studies published in English were included. Studies without scientific credibility or non‐peer‐reviewed were excluded. Animal studies and those investigating obesity grade 3 or more (BMI > 40 kg/m^2^) were also excluded as the aim of this study was not to look at severe type of obesity.

### 2.3 Data extraction

Two reviewers (FE, KR) independently extracted data from quantitative studies using the data extraction tool from JBI‐MAStARI (Aromataris & Munn, 2020). Study characteristics including specific details of included studies, population demographics, methods and outcomes of interest to the review questions were extracted from each study (Aromataris & Munn, 2020). The qualitative data were extracted independently by both reviewers (FE, KR) using the standardized data extraction tool form JBI‐QARI (The Joanna Briggs Institute, 2020).

### 2.4 Assessment of methodological quality

Included studies were evaluated independently by two reviewers (FE, KR) for methodological validity and risk of bias using the standardized critical appraisal tools from JBI‐MAStARI (Aromataris & Munn, 2020), which were specific for each article's study design. The quality scores were based on the possibility of risk of bias in the methodology, conduct, and analysis in which “Yes” represented a quality score of 1 (Oldroyd et al., 2022). The following maximum scores showed the highest quality: cross‐sectional, 8; cohort studies, 11; and qualitative, 10. For this review, methodological quality is reported; however, this did not influence inclusion or exclusion of studies.

### 2.5 Data synthesis

#### 2.5.1 Quantitative data synthesis

To examine the heterogeneity and suitability of quantitative data for meta‐analysis, a statistician (AB) was consulted. The main statistics extracted from each study were the mean BMI for individuals experiencing food security and the mean BMI for individuals with food insecurity, together with odds ratio (OR) and 95% confidence intervals (CI). When an OR was not reported, it was estimated from other data based on the methods outlined in the Cochrane handbook (Higgins et al., 2021). The Comprehensive Meta‐analysis software version 3 was used to pool effect sizes in a random‐effects meta‐analysis. A random‐effects model was applied to quantify pooled effect sizes and 95% CI. Using Tau statistics, heterogeneity was calculated. Where statistical pooling for extracted statistics (variables related to the food environments) was not possible, the results were presented in a narrative form according to the Synthesis Without Meta‐Analysis (SWiM) guidelines (Campbell et al., 2020).

#### 2.5.2 Qualitative data synthesis

An approach outlined by Thomas and Harden (2008) was used to develop thematic synthesis for qualitative data. To this end, data were firstly open‐coded using line‐by‐line coding technique (FE, CO). Then, based on similarities identified within the data, descriptive themes were developed. Finally, analytical themes were developed and were reviewed and agreed (FE, CO).

#### 2.5.3 Data synthesis for mixed‐methods synthesis

The findings of quantitative and qualitative data were aggregated according to a convergent segregated approach outlined in the JBI Reviewers' Manual for JBI Mixed Methods Systematic Reviews (Stern, Lizarondo, Carrier, et al., 2020). This included a configurative analysis approach to generate the links between the findings that represented aggregation. The finding themes from quantitative and qualitative synthesis are presented in narrative description (Campbell et al., 2020; Stern, Lizarondo, Carrier, et al., 2020).

## 3 RESULTS

### 3.1 Main characteristics of the studies

After removal of duplicates, a total of 6307 citations were found. After title and abstract screening, 149 full‐text papers were evaluated against the eligibility criteria. One more study was found from checking reference lists. One hundred and three publications were excluded at this stage. Reasons for exclusions are presented in Figure 1. The study selection flowchart is presented according to PRISMA guidelines (Moher et al., 2009; Figure 1). For the quantitative section of the review, 36 cross‐sectional studies (Bauer et al., 2012; Bruening et al., 2012; Dharod et al., 2013; Domingo et al., 2021; Gorski Findling et al., 2018; Huelskamp et al., 2021; Kaiser et al., 2019; Keenan et al., 2021; Kral et al., 2017; Leung & Villamor, 2011; Matheson et al., 2002; McCurdy et al., 2015; Mercille et al., 2012; Nettle & Bateson, 2019; Nguyen et al., 2015; Niu et al., 2021; Poulsen et al., 2019; Ro & Osborn, 2018; Robaina & Martin, 2013; Rodriguez et al., 2021; Sanjeevi et al., 2018; Santarossa et al., 2021; Shinwell et al., 2021; Smith & Richards, 2008; Vadiveloo et al., 2020; van der Velde et al., 2020; Vedovato et al., 2016; Walch & Holland, 2021; Watt et al., 2013; Webb et al., 2008; Widome et al., 2009; Wilcox et al., 2020; Wirth et al., 2020; Yau et al., 2020) and one cohort study (Benjamin‐Neelon et al., 2020) were included. Eleven studies were included for the qualitative section of the review (Bhawra et al., 2015; Byker Shanks et al., 2020; Cooksey Stowers et al., 2020; Franzen & Smith, 2009; Genuis et al., 2015; Gosliner & Shah, 2020; Holston et al., 2020; Jennings et al., 2020; Kerpan et al., 2015; Ong et al., 2021; Thompson et al., 2018).

Included studies were primarily conducted across the USA (36) (Bauer et al., 2012; Benjamin‐Neelon et al., 2020; Bruening et al., 2012; Byker Shanks et al., 2020; Cooksey Stowers et al., 2020; Dharod et al., 2013; Franzen & Smith, 2009; Gorski Findling et al., 2018; Gosliner & Shah, 2020; Holston et al., 2020; Hooper et al., 2020; Huelskamp et al., 2021; Jennings et al., 2020; Kaiser et al., 2019; Kral et al., 2017; Leung & Villamor, 2011; Matheson et al., 2002; McCurdy et al., 2015; Nettle & Bateson, 2019; Nguyen et al., 2015; Niu et al., 2021; Poulsen et al., 2019; Ro & Osborn, 2018; Robaina & Martin, 2013; Rodriguez et al., 2021; Sanjeevi et al., 2018; Santarossa et al., 2021; Smith & Richards, 2008; Vadiveloo et al., 2020; Vedovato et al., 2016; Walch & Holland, 2021; Watt et al., 2013; Webb et al., 2008; Widome et al., 2009; Wilcox et al., 2020; Wirth et al., 2020), with six in Canada (Bhawra et al., 2015; Domingo et al., 2021; Genuis et al., 2015; Kerpan et al., 2015; Mercille et al., 2012; Ong et al., 2021), four in the UK (Keenan et al., 2021; Shinwell et al., 2021; Thompson et al., 2018; Yau et al., 2020), and one in Netherlands (van der Velde et al., 2020). Twenty‐two studies were published between 2020 and 2021 (Benjamin‐Neelon et al., 2020; Byker Shanks et al., 2020; Cooksey Stowers et al., 2020; Domingo et al., 2021; Gosliner & Shah, 2020; Holston et al., 2020; Hooper et al., 2020; Huelskamp et al., 2021; Jennings et al., 2020; Keenan et al., 2021; Niu et al., 2021; Ong et al., 2021; Rodriguez et al., 2021; Santarossa et al., 2021; Shinwell et al., 2021; Vadiveloo et al., 2020; van der Velde et al., 2020; Walch & Holland, 2021; Wilcox et al., 2020; Wirth et al., 2020; Yau et al., 2020), 14 studies were published between 2015 and 2019 (Bhawra et al., 2015; Genuis et al., 2015; Gorski Findling et al., 2018; Kaiser et al., 2019; Kerpan et al., 2015; Kral et al., 2017; McCurdy et al., 2015; Nettle & Bateson, 2019; Nguyen et al., 2015; Poulsen et al., 2019; Ro & Osborn, 2018; Sanjeevi et al., 2018; Thompson et al., 2018; Vedovato et al., 2016) and the remaining were published between 2002 and 2014 (Bauer et al., 2012; Bruening et al., 2012; Dharod et al., 2013; Franzen & Smith, 2009; Leung & Villamor, 2011; Matheson et al., 2002; Mercille et al., 2012; Robaina & Martin, 2013; Smith & Richards, 2008; Watt et al., 2013; Webb et al., 2008; Widome et al., 2009). Total sample size was 63,152, ranging from 10 (Cooksey Stowers et al., 2020) to 8333 (Nguyen et al., 2015) in individual studies. Thirty‐two studies considered adult samples (>18 years) (Bauer et al., 2012; Bhawra et al., 2015; Bruening et al., 2012; Byker Shanks et al., 2020; Cooksey Stowers et al., 2020; Dharod et al., 2013; Domingo et al., 2021; Franzen & Smith, 2009; Gosliner & Shah, 2020; Holston et al., 2020; Huelskamp et al., 2021; Kaiser et al., 2019; Keenan et al., 2021; Leung & Villamor, 2011; McCurdy et al., 2015; Mercille et al., 2012; Nettle & Bateson, 2019; Nguyen et al., 2015; Ong et al., 2021; Ro & Osborn, 2018; Robaina & Martin, 2013; Rodriguez et al., 2021; Sanjeevi et al., 2018; Santarossa et al., 2021; Shinwell et al., 2021; Vadiveloo et al., 2020; van der Velde et al., 2020; Vedovato et al., 2016; Walch & Holland, 2021; Watt et al., 2013; Webb et al., 2008; Wilcox et al., 2020; Yau et al., 2020), 11 studies focused on children or adolescents (Benjamin‐Neelon et al., 2020; Genuis et al., 2015; Gorski Findling et al., 2018; Jennings et al., 2020; Kerpan et al., 2015; Kral et al., 2017; Matheson et al., 2002; Poulsen et al., 2019; Smith & Richards, 2008; Widome et al., 2009; Wirth et al., 2020) and three studies included both children/adolescents and adults (Hooper et al., 2020; Niu et al., 2021; Thompson et al., 2018). Twenty‐three quantitative studies directly measured anthropometric indices (Bauer et al., 2012; Benjamin‐Neelon et al., 2020; Dharod et al., 2013; Domingo et al., 2021; Hooper et al., 2020; Kral et al., 2017; Matheson et al., 2002; Mercille et al., 2012; Nettle & Bateson, 2019; Nguyen et al., 2015; Niu et al., 2021; Poulsen et al., 2019; Robaina & Martin, 2013; Rodriguez et al., 2021; Sanjeevi et al., 2018; Santarossa et al., 2021; Smith & Richards, 2008; Vedovato et al., 2016; Watt et al., 2013; Widome et al., 2009; Wilcox et al., 2020; Wirth et al., 2020) and 14 studies used self‐reported measures (Bruening et al., 2012; Gorski Findling et al., 2018; Huelskamp et al., 2021; Kaiser et al., 2019; Keenan et al., 2021; Leung & Villamor, 2011; McCurdy et al., 2015; Ro & Osborn, 2018; Shinwell et al., 2021; Vadiveloo et al., 2020; van der Velde et al., 2020; Walch & Holland, 2021; Webb et al., 2008; Yau et al., 2020). A summary of all included quantitative and qualitative studies are presented in Tables 1 and 2, respectively.

**TABLE 1:** Characteristics of the included studies and^a^ quality assessment, quantitative component

Author (date), country	Study type (size) and population	Variables measured and measuring instruments	Key findings	QA score
Yau et al. (2020) UK	Cross‐sectional *N* = 2551 among full analytic sample (*n* = 1949 for BMI sub‐sample) Ages 18–64 Gender: All	Food insecurity: 10‐item USDA Adult Food Security Survey Module (AFSSM) Weight status: Self‐reported height and weight (then BMI kg/m^2^ was calculated) Other variables: Fruit and vegetable intake was measured using questions adopted from the validated 2017 Behavioral Risk Factor Surveillance System fruit and vegetable intake module Self‐rated the healthiness of diet	Outcome 1: In BMI sub‐sample, 21.8% of participants were food insecure. Adults with food insecurity had higher odds of overweight compared to adults with food security (OR = 1.32, 95% CI = 1–1.75). (Note: in this study, participants were only categorized as: not overweight or overweight. Other participants had missing height and/or weight values or were excluded because of an extreme BMI value (<14 or >48). The prevalence of obesity was not therefore reported). This association was stronger in women aged 40–49 years than in men Outcome 2: Compared to food secure group, adults with food insecurity had higher odds of reporting unhealthy diets (OR = 1.65, 95% CI = 1.31–2.09) Outcome 3: Compared to food secure group, adults with food insecurity had lower odds of eating fruits and vegetables (OR = 0.59, 95% CI = 0.47–0.74 and OR = 0.68, 95% CI = 0.54–0.86, respectively), but had higher odds for consuming fruit juice (OR = 1.39, 95% CI = 1.10–1.75) Outcome 4: Interactions by sex, ethnicity and age were reported for odds of food insecurity; the associations between food insecurity and vegetable and fruit juice intake frequency were significant in women (OR = 1.66, 95% CI = 1.21–2.28) but were not in men (OR = 0.53, 95% CI = 0.39–0.73). In terms of ethnicity, white British adults with food insecurity had higher odds of above median fruit juice intake frequency (OR = 1.5, 95% CI = 1.16–1.93), however, adults who were black and experienced food insecurity had lower odds of above median fruit juice intake (OR = 0.11, 95% CI = 0.02–0.62). Age altered the association between food insecurity and fruit intake; the adjusted odds of fruit intake were significantly lower in those adults with food insecurity across all age groups except those aged 40–49 and 60–64 years old, compared to adults with food security Outcome 5: In terms of socio‐demographic correlates of food insecurity, the odds of it were higher among those who reported their ethnicity as white other or mixed (OR = 2.04, 95% CI = 1.04–3.99; OR = 2.32, 95% CI = 1.02–5.27, respectively) compared to white British. Compared to those living alone, the odds of food insecurity were higher for those living with children, especially among single‐parent households (OR = 2.32, 95% CI = 1.19–3.70). Compared to non‐student groups, full‐time students had higher odds of food insecurity (OR = 3.25, 95% CI = 2.01–5.18). Compared to those with high education, odds of food insecurity were higher among adults with low education. Compared to those who reported making ends meet was easy, those adults who reported difficulty in making ends meet had the strongest associations with food insecurity (OR = 19.76, 95% CI = 13.78–28.34)	8
Keenan et al. (2021) UK	Cross‐sectional *N* = 604 Ages 18–75 Gender: All	Food insecurity: 10‐item USDA Adult Food Security Survey Module (AFSSM) Weight status: Self‐reported height and weight (then BMI kg/m^2^ was calculated). Other variables: A short food frequency questionnaire, taken from the Yorkshire Health Survey, was used to assess diet quality. Eating to cope: coping subscale from the Palatable Eating Motives Scale was used	Outcome 1: The level of moderate food insecurity was 21.5% Outcome 2: Mean BMI (*SD*) was 29.19 (7.86) kg/m^2^. Near 38% of the sample were classified with obesity, near 29% with overweight, near 3% as underweight, and 30.8% with healthy weight Outcome3: Indirect association between food insecurity and higher BMI was significant via greater distress and eating to cope (beta coefficient [*SE*]: 0.13 [0.03]; *p* < .001, 95% CI = 0.07–0.20) Outcome 4: Direct association between food insecurity and poorer diet quality was significant, but this relationship was not explained by distress and eating to cope (beta coefficient [*SE*]: −0.47 [0.10]; *p* < .001, 95% CI = −0.65 to 0.28) Outcome 5: Near 23% ate five or more portions of fruit and vegetables a day	8
Shinwell et al. (2021) UK	Cross‐sectional *N* = 394 Mean age (*SD*) 29–38 (3–13) Gender: All	Food insecurity: 6‐item Household Food Security Survey Short Form module (HFSSM) developed by the US National Centre for Health Statistics (NCHS) Weight status: self‐reported height and weight (then BMI kg/m^2^ was calculated) Other variables: 1. Dietary intake data that were collected using Intake24 (an open source, on‐line, self‐completed dietary recall system)	Outcome 1: There was no significant association between food insecurity status and BMI in the UK data (this study compared patterns found in the UK sample with those from the USA National Health and Nutrition Examination Survey [NHANES 2013–2014 datasets]), even considering an expected interaction with gender (*p* = .91). Therefore, the authors did not examine mediation of the food insecurity‐BMI association by food consumption variables Outcome 2: Adults with food insecurity consumed significantly more carbohydrate but less protein than those with food security. However, there was no significant difference in total energy intakes or relative fat and fiber intakes by food security status Outcome 3: Adults with food insecurity had larger and more variable time gaps between consumption events. However, they had a significantly smaller and less variable number of foods per consumption events	8
Niu et al. (2021) USA	Cross‐sectional *N* = 1328 Ages 14–21 Gender: Female	Food insecurity was assessed using the following questions: (1 = sometimes or often do not have enough to eat in the household; 0 = enough to eat) Weight status: Directly measured weights and heights. Other variables: 1. Neighborhood social characteristics (percent sugary drinks, percent no fruits and vegetables)	Outcome 1: Those with food insecurity had a higher BMI than girls with food security (*b* = 1.53, 95% CI = 0.19–2.87, *p* < .05) Outcome 2: Five distinct neighborhood profiles in New York City were identified: High Structural/High Social Advantage, Moderate Advantage/Low Crime, Low SES (Socioeconomic Status)/High Activity, Low SES/High Social Advantage, and High Disadvantage Outcome 3: Compared to living in High Disadvantage neighborhoods, living in Low‐SES/High‐Activity neighborhoods was found to be associated with a lower BMI at 22 years (*b* = 1.21, 95%CI = −2.35, − 0.06, *p* < .05). Also, a slower rate of change in BMI was found among those living in Low‐SES/High‐Activity neighborhoods (95% CI = 0.45, 0.02, *p* = .07). Other neighborhood profiles were not significantly different from the reference profile	8
Huelskamp et al. (2021) USA	Cross‐sectional *N* = 547 Ages Adults (college students) Gender: All	Food insecurity: 11‐item USDA Adult Food Security Survey Module Weight status: self‐reported height and weight (then BMI kg/m^2^ was calculated) Other variables: A questionnaire to identify strategies used in the last 12 months to access food to indicate food (in) security (such as Purchased cheap and/or processed foods) A questionnaire to ask about lifestyle habits A question asked, “What would currently help you improve your food situation?”	Outcome 1: Most of students (63%) reported they struggle with some level of food insecurity. Near 7% of them reported they sometimes/often did not have enough to eat. Another 56% indicated they had enough food, but not always the kinds of food they wanted Outcome 2: A significant but weak positive association between food insecurity status and BMI was found (*r*_s_ = .1026, *p* = .05) Outcome 3: Obesogenic behaviors were more prevalent among those with food insecurity. This group was 13% more likely to purchase cheap processed food (such as frozen pizza or Ramen noodles), 17% more likely to eat more than normal when food was plentiful, and 24% more likely to eat less healthy meals (to eat larger quantities of food) Outcome 4: Only 9% of those with food insecurity and 5% with food security status reported obtaining food from a foodbank (14% of respondents rated access to an on‐campus food pantry as a helpful form of support) (*p* < .05) Outcome 5: The largest proportion of students ranked employment as a helpful form of support, followed by “learn how to eat healthy,” “learn how to make a budget,” “more financial aid at school,” and “learn to cook” (*p* < .05)	6
Walch and Holland (2021) USA	Cross‐sectional *N* = 148 Ages Adults aged 18 and over (clients from the largest food pantry in Alaska) Gender: All	Food insecurity: 10‐item USDA Adult Food Security Survey Module (AFSSM) Weight status: self‐reported height and weight (then BMI kg/m^2^ was calculated) Other variables: 1. Frequency of visits to food pantries	Outcome 1: Most of participants had overweight or obesity (70%) and experienced food insecurity (88.4%) Outcome 2: Between weight and food security status, no significant associations were found Outcome 3: Most clients (65%) visited the pantry every month and 72% received food from at least one other pantry over the past year	8
Rodriguez et al. (2021) USA	Cross‐sectional *N* = 92 Ages Adults aged 18 and over (clients from local food pantries) Gender: Female	Food insecurity: 18‐item US Household Food Security Survey Module (HHFSM) Weight status: Directly measured weights and heights Other variables: A 27‐item self‐report measured survey to assess individuals' intakes of dietary fats, fruit, and vegetable consumption over last 30 days	Outcome 1: Compared to participants with food security, those with food insecurity showed higher BMI (*t* (90) = −2.15, *p* = .03, *d* = 0.5) and percent of body fat (t (90) = −2.22, *p* = .03, *d* = 0.5) Outcome 2: They also consumed fewer servings of fruits and vegetables (t (90) = −2.22, *p* = .03, *d* = 0.5) Outcome 3: Those with FI reported annual incomes less than $20,000 compared to those with food security (χ^2^ (1) = 7.65, *p* = .006)	8
Domingo et al. (2021) Canada	Cross‐sectional *N* = 3681 Ages adults ≥ 19 Gender: All	Food insecurity: 18‐item US Household Food Security Survey Module (HHFSM) Weight status: Measured and self‐reported height and weight Other variables: Cost and affordability of healthy eating within each community were measured using 67‐basic food items from Health Canada's 2008 National Nutritious Food Basket Tool Distance to service centre from each community was assessed from road access to a service centre (access to suppliers etc)	Outcome 1: Food insecurity was prevalent among 46% of households Outcome 2: Predictors of food insecurity were those who receive social assistance, being female, having children, having <10 years of education, age between 19 and 30 years, living in Alberta, and having no year‐round road access into community (*p* < .001) Outcome 3: Those who resided in households with marginal food insecurity had the highest rates of obesity (for both females 57%; and males 55%, *p* < .05). Rates of obesity were lowest for households with severe food insecurity	8
Santarossa et al. (2021) USA	Cross‐sectional *N* = 1100 Ages (*SD*) 67.3 (16.1) Gender: All	Food security: 18‐item US Household Food Security Survey Module (HHFSM) Weight status: Measured height and weight Other variables: The availability, price, and quality of healthy foods available in retail food stores relative to less healthy choices were assessed using 11‐item Nutrition environment measures survey in stores (NEMS‐S) tool	Outcome 1: Participants with food insecurity were more likely to live in areas with: higher rates of public assistance or food stamps/SNAP (*p* = .014), higher rates of no vehicle access (*p* = .022), lower median household incomes (*p* = .014), and higher unemployment rates (*p* = .014) Outcome 2: After controlling for socio‐demographic factors associated with food insecurity, NEMS‐S score in a 2‐mile radius was significantly associated with food insecurity (where those with food insecurity had on average 0.39 higher NEMS‐S scores). This suggested that those with food insecurity did not live further away from healthier grocery stores, and this was not also modified by ecological measures of vehicle access. It was concluded that those with food insecurity in Detroit are likely to be limited by contextual factors (and not by their neighborhood or physical access to healthy grocery stores) Outcomes 3: Those with food insecurity were younger at screening (*p* < .001) and were less likely to be married (*p* < .001)	8
Wirth et al. (2020) USA	Cross‐sectional *N* = 3019 Ages 2–17 years Gender: All	Food insecurity: The Hunger Vital Sign (HVS) screener tool Weight status: Measured height and weight Other variables: To identify food access, the 2015 USDA food access research atlas (a national data set that provides food access data for populations) was used	Outcome 1: Those children with food insecurity had a lower mean BMIz than those with food security (0.65 vs. 0.82, *p* = .01) Outcome 2: A smaller percentage of children with food insecurity had obesity compared to those with food security (17.4% vs. 26.2%, *p* = .02). A larger percentage of children with food insecurity had healthy weight compared to children experiencing food security (63.5% vs. 55.1%, *p* = .02) Outcome 3: Obesity was negatively associated with household food insecurity (OR = 0.55, 95% CI = 0.35–0.86, *p* < .01) Outcome 4: Living in a neighborhood with low access to supermarkets were not associated with household food insecurity Outcome 5: Children with food insecurity lived in neighborhoods with a higher proportion of households that lived below the federal poverty level (FPL) than those with food security (mean difference: 3.9%, 95% CI = 1.6, 6.3)	8
van der Velde et al. (2020) Netherlands	Cross‐sectional *N* = 250 Ages 18 years of age or older Gender: Predominantly women	Food insecurity: 18‐item US Household Food Security Survey Module (HHFSM) Weight status: Self‐reported height and weight Other variables: Dutch Healthy Diet Food Frequency Questionnaire (DHD‐FFQ) was used to assess dietary intakes and to construct diet quality scores Information on food bank use was also collected	Outcome 1: Prevalence of food insecurity was 26% (of which 18.2% experienced low food security and 7.8% experienced very low food security) Outcome 2: Obesity prevalence was higher for those with very low food security (58%) compared to those with high food security (24%). In total, 43% of those with food insecurity were obese while 25% of those with food security were obese Outcome 3: Compared to those with food security, those living with food insecurity reported more often to have an income below the basic needs budget, to have a lower educational level, and reported less often currently employed Outcome 4: Findings from this study suggested an association between food insecurity and obesity. In the unadjusted model, food insecurity was associated with obesity (OR = 2.49, 95% CI = 1.16, 5.33), but not with overweight (OR = 1.15, 95% CI = 0.54, 2.45) Outcome 5: Those experiencing food insecurity were 2.49 times more likely to be obese than those with food security (95% CI = 1.16, 5.33; *p* = .019) Outcome 6: After adjustment for other covariates, the association between food insecurity and obesity was partially mediated by diet quality (proportion mediated: − 18.6%)	8
Hooper et al. (2020) USA	Cross‐sectional *N* = 2285 Mean age (*SD*): 14·4 (10–22) Gender: All	Food insecurity: Six‐item US Household Food Security Survey Module Weight status: Measured height and weight Other variables: The home food environment was assessed using the Project F‐EAT survey The EAT 2010 survey (a 235‐item self‐report instrument) assessed factors relevant to weight status and weight‐related behaviors	Outcome 1: Near 40% of adolescents experienced household food insecurity over the past year, and near 40% were overweight Outcome 2: Those with food insecurity had had higher prevalence of overweight (42·3%) compared with those with food security (37·9%) (*p* = .039). They also had lower breakfast consumption (*p* = .005) and greater use of unhealthy weight control behaviors (*p* < .001)	
Vadiveloo et al. (2020) USA	Cross‐sectional *N* = 3961 Mean ages (*SE*): 50.6 (0.53) Gender: All	Food security: A modified USDA 30‐day adult food security scale Weight status: Self‐ reported height and weight (then BMI kg/m^2^ was calculated) Other variables: 3 USDA Food Acquisition and Purchasing data were used to record (i) Food at home and (b) Food away from home 4 The healthy eating index (HEI‐2015) was used to assess diet quality of food at home acquisitions 5 Participation in SNAP	Outcome 1: Prevalence of food insecurity was 14.0%, and near 13% of respondents participated in SNAP Outcome 2: Mean HEI‐2015 scores were 54.7 (out of 100) and the scores varied across all sociodemographic exposures (*p* < .05) Outcome 3: Those with food security who were non‐SNAP had higher HEI score (53.9) than those with food insecurity who were SNAP‐users (50.3) (*p* = .007) Outcome 4: HEI‐2015 score was significantly higher for non‐SNAP households without obesity than other households (*p* < .05)	8
Wilcox et al. (2020) USA	Cross‐sectional *N* = 527 Mean ages (*SE*) 52.33 (14.19) Gender: All	Food insecurity: USDA Household Food Security questionnaire Weight status: Measured height and weight Other variables: Food desert residence was assessed using the USDA's Food Access Research Atlas The Healthy Eating Index 2010 (HEI‐2010) was used to assess diet quality To assess dietary intakes, one 24‐h dietary recall was taken Receipt of SNAP in the past year	Outcome 1: Majority of participants were overweight or obese (23.43% and 55.75%, respectively). Sixty‐three percent of participants had low or very low food security Outcome 2: Those with food insecurity were less likely to be below the dietary guidelines for carbohydrates than those with food security (46% vs. 35%, OR = 1.66, CI: 1.12–2.46) Outcome 3: Those with food insecurity were more likely than those with food security to be above the guidelines for total fat (51% vs. 40%; OR 1.58, 95% CI: 1.07–2.33) Outcome 4: Those with food insecurity were also less likely than those with food security to meet the guidelines for whole grains (8% vs. 15%) Outcome 5: The mean diet quality score was 49 (out of 100). Diet quality for those experiencing food security was significantly higher than those with food insecurity (*p* = .03) Outcome 6: More than 82% of participants lived in a urban food desert (census tracts of low income and low access to supermarkets. However, the results of the linear regression models indicated that food desert residence was not related to diet quality Outcome 7: Near 64% of participants received SNAP benefits Outcome 8: It was found that lower household income was associated with being below the dietary guidelines for carbohydrates, fruits, vegetables, and above the guideline for sodium	8
Benjamin‐Neelon et al. (2020) USA	Longitudinal (cohort) *N* = 666 Ages 3–12 months Gender	Food security: 18‐item US Household Food Security Survey Module (HHFSM) Weight status: Measured height and weight Other variables: Participation in the Special Supplemental Nutrition Program for Women, Infants, and Children (WIC) Participation in Supplemental Nutrition Assistance Program (SNAP)	Outcome 1: Interactions between food security status and participation in WIC and/or SNAP were not significant (*p* > .05) Outcome 2: Compared with those infants from high food security households, those from very low food security households had higher BMI z scores (0.18 U; 95% CI = 0.01–0.35; *p* = .04), and greater odds of being at risk for overweight (OR = 1.55; 95% CI = 1.14–2.10; *p* = .005) Outcome 3: The odds of being at risk for overweight was higher for infants from low food security households (OR = 1.72; 95% CI = 1.17–2.10; *p* = .005)	11
Nettle and Bateson (2019) USA	Cross‐sectional *N* = 2798 Age > 18 Gender: Females	Food insecurity: Adult questions of the standard USDA household Food Security Survey Module Weight status: Measured height and weight (then BMI kg/m^2^ was calculated). Other variables: 24‐h food recall data	Outcome 1: Those with food insecurity had higher BMIs (Mean BMI = 31.13, *SD* = 8.86) than those with food security (Mean BMI = 28.77, *SD* = 7.37, *p* < .001) Outcome 2: Compared to those with food security, 15% of difference in BMI in those with food insecurity was accounted for by their lower diversity of foods, lower fiber intakes, and more variable time gaps between their eating. They also consumed more carbohydrate and less protein	8
Ro and Osborn (2018) USA	Cross‐sectional *N* = 5957 Ages 18–65 Gender: All	Food insecurity or food poverty: 6‐item USDA Household Food Security Survey Module Weight status: Self‐ reported height and weight (then BMI kg/m^2^ was calculated). Other variables: Neighborhood Fresh Produce Environment Participation in Nutrition assistance programs was assessed by receipt of food stamp benefits	Outcome 1: Latinas with severe food insecurity were more affected by overweight or obesity (79.1%) than women with food security or with low food insecurity (70.7%) (OR = 1.50, 95% CI = 1.03–2.19) Outcome 2: Regarding the neighborhood fresh produce environment, women with severe food insecurity perceived significantly lower neighborhood availability (72.5%) and affordability (46.5%) of fresh produce in comparison to women who did not experience severe food insecurity Outcome 3: Males with severe food insecurity reported a lower percentage of neighborhood availability (75.9%) and affordability (40.9%) in comparison to males who had food security Outcome 4: Neighborhood affordability of fresh produce was statistically associated with overweight/obesity. If women with severe food insecurity were able to afford fresh produce in their neighborhood, then they had lower odds of obesity (OR = 0.70, 95% CI = 0.53–0.93)	8
Bruening et al. (2012) USA	Cross‐sectional *N* = 2095 Ages 32.7–50.2 Gender: All	Food insecurity or food poverty: 6‐item US Household Food Security Survey Module (HHFSM) Weight status: Self‐reported height and weight (BMI was then calculated) Other variables: Home food environment: To assess perceptions of FV access, addressing cost, variety, and quality of produce	Outcome 1: Near 39% of participants had food insecurity, and about 13% experienced very low food security. Outcome 2: Parents with food insecurity reported significantly poorer eating behaviors than did parents with food security. Parents with food insecurity consumed breakfast less frequently but ate more serving of SSB Outcome 3: The home food environment was poorer in households experiencing food insecurity than in households with food security. Parents with food insecurity consumed more fast‐food and SSB at family meals. However, they ate less healthy items such as green salad, vegetables, and fruit (95% CI = 2.5, 10.3) Outcome 4: Large differences were reported in perceived access to fruits and vegetables according to food security status. Near 40% of parents with food insecurity agreed that fruits were too expensive to purchase compared with near 14% of parents with food security (95% CI = 21.5, 30.6)	8
McCurdy et al. (2015) USA	Cross‐sectional, correlational design *n* = 166 Mean age 30.1 ± 7.2 Gender: Female (mothers)	Food insecurity or food poverty: 18‐item the Food Security Core Module (FSCM) Weight status: Self‐reported height and weight Other variables: Participation in food assistance programs Supermarket Use Food Shopping Practices to rate use of shopping practices to stretch food dollars	Outcome 1: 67% of participants experienced overweight or obesity, and 42% had household food insecurity Outcome 2: 80% of participants used benefits from government food assistance programs (such as SNAP and/or WIC benefits). Outcome 3: Participation in food assistance programs (*p* < .05), more frequent use of food shopping practices to stretch food dollars (*p* = .04), and household food insecurity (*p* < .04) were positively associated with maternal BMIs were positively associated. However, participation in WIC or SNAP programs and number of weekly shopping visits to supermarkets were not associated with maternal BMI	8
Sanjeevi et al. (2018) USA	Cross‐sectional *N* = 152 Ages 19–50 Gender: Female	Food insecurity or food poverty: 10 questions in the U.S. Adult Food Security Scale (The 10‐item U.S. adult food security survey) Weight status: A digital weighing scale and stadiometer were used to measure height and weight. BMI then calculated. Other variables: Multi‐dimensional home environmental scale (MHES)	Outcome 1: The average BMI was 29.6 kg/m^2^. According to BMI, the proportions of healthy weight, overweight, and obesity were 28.9%, 36.2% and 34.9%, respectively Outcome 2: The proportions of women who experienced food security and food insecurity were near 40% and over 60%, respectively. Food insecurity was positively associated with BMI (beta coefficient = 0.213; *p* < .01). The average BMI for food security and food insecurity was near 28 and 31 kg/m^2^, respectively Outcome 3: BMI was significantly associated with availability of unhealthy food at home (beta coefficient = −0.227, *p* = .02) Outcome 4: A significant difference was reported for availability of unhealthy food at home by food security status. Groups with food security scored higher than groups with food insecurity for the availability of unhealthy food at home by 13.8% (*p* < .01) Outcome5: The association between food insecurity and obesity was partially mediated by home food environment (beta coefficient: 0.19, 95% CI: 0.01–0.42, *p* < .05)	7
Vedovato et al. (2016) USA	Cross‐sectional *N* = 298 Age adult caregiver–child (10–14 years old) dyads Gender: All	Food insecurity or food poverty: The USDA's 18‐item Household Food Security Scale Weight status: Directly measured weights and heights. Other variables: Food source destinations Four subscales were created to indicate different beliefs and attitudes about healthy food. These included affordability, convenience, importance, and taste	Outcome 1: Near 42% of families had different levels of food insecurity, with more than 12% had moderate or severe hunger. Higher proportion of adults and children classified as overweight were found among low‐income African‐American households with food security. But higher rates of adults and children classified as obese were found among those with food insecurity with hunger. The prevalence of overweight and obesity for those living in families with food insecurity and hunger was near 89% for adults and 40·5% for children Outcome 2: There was no significant association between food source use pattern and excess body weight Outcome 3: Households with food security had a higher level of agreement with healthy food to be affordable, compared with both food insecurity groups. Respondents who experienced food insecurity without hunger considered healthy foods less accessible and less convenient to buy in the neighborhood and to prepare at home (*p* < .05)	8
Robaina and Martin (2013) USA	Cross‐sectional (Baseline data from a longitudinal assessment of a community‐based program) *N* = 212 Age > 18 years Gender: All	Food insecurity or food poverty: 18‐item USDA Household Food Security Module (HFSM) Weight status: Height and weight were measured using a stadiometer and digital medical scale. Other variables: Participants reported how frequently they use food pantries or soup kitchens, and whether they receive SNAP, WIC The Block Food Frequency Screener to measure diet quality. Intakes of fruit, vegetables, and fiber were reported	Outcome 1: Near 16% of the population experienced food security, 33.5% had low food security, and the remaining over half of the sample reported very low food security (i.e., food insecurity with hunger) Outcome 2: The average BMI was 29.5 kg/m^2^ (*SD*: 7.0). Almost 32% of the sample were affected by overweight, and 29.8% experienced obesity Outcome 3: Participants used food pantries on a long‐term basis, with 62.5% going at least once per week. Almost half (44%) of participants consumed meals at a soup kitchen. Over half of participants (57%) received SNAP Outcome 4: Participants experiencing food security were twice as likely to consume fruit, vegetables, and fiber than participants with food insecurity (OR = 2.3, 95% CI: 1.1, 5.2, *p* = .05)	7
Webb et al. (2008) USA	Cross‐sectional *N* = 435 Age ≥ 18 Gender: All	Food insecurity or food poverty: – 18‐item USDA HFSM scale was used for households with children. – The adult‐specific 10‐item subset was used for households without children Weight status: Self‐reported height and weight Other variables: Participation in 3 government‐sponsored nutrition programs	Outcome 1: 51% of respondents experienced overweight, and 25% were affected by obesity Outcome 2: 30% of households reported having food insecurity during the year prior to the survey and nearly 45% of them had severe food insecurity with hunger Outcome 3: Cases who categorized as food insecure or who had food insecurity with hunger experienced significantly higher BMI than those classified as food secure (*p* < .01) Outcome 4: Respondents who reported their food supplies did not last, those who were not able to afford balanced meals, those who cut their meal sizes and consumed less than their required need had significantly higher BMI than those who never having those experiences (*p* < .01) Outcome 5: Respondents who participated in the FSP, WIC and free/reduced‐price school meals over the 12 months before to the survey had significantly higher BMI than those who did not receive benefits from government (*p* < .01) Outcome 6: Those who acquired food from charitable sources such as food banks or soup kitchens had significantly higher BMI (*p* < .01). Shopping at convenience stores (*p* = .04) and higher consumption of fast foods was associated with higher BMI (*p* < .01). There was no difference in BMI based on different use of supermarkets, ethnic grocery stores, or farmers' markets	8
Bauer et al. (2012) USA	Cross‐sectional (baseline survey of RCTs) *N* = 432 Age Parents or caregivers of kindergarten‐aged children Gender: All	Food insecurity or food poverty: The 6‐item short form of the Household Food Security Scale Weight status: Measured height using a portable stadiometer. Measured weight using Tanita scales (children: age‐ and gender‐specific BMI percentiles; adults: BMI for adults) Other variables: Home food availability was measured Family food practice: Parents were asked about the frequency of fast‐food trips per week Barriers to healthful food at home were reported	Outcome 1: Almost 40% of parents and their households had food insecurity over the last year. More than 10% of households had very low food security. Families with a lower total household income and those unemployed had more proportion of food insecurity Outcome 2: No significant differences were observed in either children's or parents' weight status by food security status Outcome 3: Youths from families who had very low food security ate hot food or ready‐made food from a convenience store or gas station more than twice as did youths from households who were food secure (*p* = .002). Youths who had food insecurity reported to consume pizza and fried chicken more often than did youths with food security Outcome 4: No differences were observed in households' home food availability or frequency of families' fast‐food trips according to food security status Outcome 5: Parents who had food insecurity were most likely to report there was little variety of fruits and vegetables where they buy groceries (*p* = .003). They were more likely to agree that where they buy groceries the fruits and vegetables had poor condition (*p* = .03)	6
Leung and Villamor (2011) USA	Cross‐sectional adult *n* = 7741 (18–≥70 years) caregiver–child (10–14 years old) dyads Gender: All	Food insecurity or food poverty: The USDA Household Food Security Survey Module: 6‐Item Short Form Weight status: BMI from self‐reported height and weight data Other variables: Participation in government nutrition assistance programs Dietary information	Outcome 1: The prevalence of obesity was 27·4% Outcome 2: The incidence of obesity was 30% higher in those who participated in SNAP (95% CI: 6%, 59%; *p* = .01) than in those who did not participate. This association was more evident among males than among females Outcome 3: Participation in SSI was associated with 50% higher prevalence of obesity compared with those who did not participate Outcome 4: Soda consumption was also higher among those who participated in SNAP and SSI than those nonparticipants of any program	8
Kaiser et al. (2019) USA	Cross‐sectional N301 Mean age: 44.74 Gender: All	Food insecurity or food poverty: 6‐item USDA Household Food Security Module Weight status: Self‐reported BMI Other variables: – Food environment was assessed using families' perception of different food accessibility within their neighborhood	Outcome 1: 37.5% of participants were as affected by obesity. Participants lived in food insecurity areas reported higher prevalence of obesity Outcome 2: Food assistance users reported higher prevalence of obesity Outcome 3: Prevalence of overweight/obesity was associated with perceived farmers' market access related to lower	8
Nguyen et al. (2015) USA	Cross‐sectional *N* = 8333 Age adult caregiver (10–14 years old) dyads Gender: All	Food insecurity: The NHANES Food Security Survey Module questionnaires Weight status: Objectively measured BMI Other variables: – Participation in the SNAP program during last 12 months	Outcome 1: BMI was significantly higher among those who had food insecurity and this group were more likely to have obesity than those who had food security (38.4% vs. 33.7%; *p* < .01) Outcome 2: Even though participation in SNAP and food insecurity each independently increased risk of higher BMI and higher chance of obesity, the combined association of SNAP receipt and food insecurity decreased BMI across all groups of food insecurity by 9% (*p* < .05)	7
Dharod et al. (2013) USA	Cross‐sectional study *N* = 195 Age, years (mean ± *SD*) 33.60 ± 8.26 Gender: Female	Food insecurity or food poverty: 10‐item Radimer/Cornell Hunger Scale Weight status: Measured height and weight Other variables: Participation in SNAP and other food assistance programs Dietary Intake was measured using a short FFQ to estimate how often food items from FV, grains, beans/lentils, meats, eggs, and dairy were eaten	Outcome 1: 41% participants had overweight (BMI = 25–29.9) and 24% had obesity (BMI ≥ 30) Outcome 2: 67% of participants had food insecurity. 23% of households who had food insecurity reported severe food insecurity or child hunger Outcome 3: Majority of participants used SNAP and WIC program benefits Outcome 4: There was significant positive association between food insecurity and higher BMI. Those who had food insecurity were almost 3 times more likely to experience overweight or obesity compared to females who had food security (OR: 2.66; CI: 1.25–5.69; *p* = .01) Outcome 5: Intakes from fruit, vegetables, and beans groups for at least once a day was less common among participants with overweight/obesity than in individuals with normal weight (*p* ≤ .05) Outcome 6: Families who had severe food insecurity or child hunger reported greater intakes of eggs (OR: 21.20; CI: 7.83–57.34; *p* < .001) Outcome 7: Food security was also predicted based on daily intakes of fruits and vegetables. When participants consumed leafy green vegetables at least once a day, the odds of food insecurity decreased by 70%–80% (OR: 0.02; CI: 0.08–0.51; *p* < .001)	8
Mercille et al. (2012) Canada	Cross‐sectional *N* = 107 (the total is 99) Age 18–65 (an average age of 38 years) Gender: Female	Food insecurity or food poverty: 18‐item USDA module survey Weight status: BMI was measured Other variables: Self‐efficacy for food preparation data. Two self‐efficacy scores were used to calculate healthy food preparation and food preparation in general Household food supplies, grocery shopping practices, access to traditional food, and perceptions of fruit and vegetable supplies in local stores were also reported	Outcome 1: The average BMI was high (mean BMI: 33.7, *SD*: 6.7) Outcome 2: Approximately 50% of households experienced food insecurity; 39% reported moderate food insecurity and 9% experienced severe food insecurity Outcome 3: Most households shopped at a supermarket located 145 km or more from their homes. Participants perceived negatively about their local grocery stores as they did not usually carry fresh FV, and it was used mainly as a backup Outcome 4: Among BMI categories, only women with severe obesity had less confidence in their healthy food preparation skills (beta coefficient: −0.23, *p* = .03) Outcome 5: The association between household food insecurity and the general food preparation score was not significant. However, severe household food insecurity and both self‐efficacy scores were inversely associated (beta coefficient: −0.25, *p* = .01) Outcome 6: No association was found between self‐efficacy scores and grocery shopping practices and access to traditional food. Lack of availability was reported as a reason for not buying fruits and vegetables locally and this was positively associated with self‐efficacy in healthy food preparation (beta coefficient: 0.29, *p* < .01)	6
Gorski Findling et al. (2018) USA	Cross‐sectional *N* = 3748 from 1942 households Age 2–18 years Gender: All	Food insecurity or food poverty: USDA's 30‐day Adult Food Security Scale Weight status: Self‐reported height and weight for calculating BMI. Other variables: Neighborhood food access Alternative neighborhood food access Household food purchases and acquisitions The mean of sugary beverage spending Household participation in SNAP and WIC	Outcome 1: Greater neighborhood access to combination grocery or other stores was correlated with higher obesity prevalence for youths overall and those participated in SNAP. Odds of childhood overweight/obesity were higher with greater access to combination grocery/other stores overall (OR: 1.10, 95% CI: 1.03–1.17) and for youths in SNAP (OR: 1.14, 95% CI: 1.05–1.24) Outcome 2: youths in SNAP households were reported to have higher odds of overweight/obesity with greater access to combination grocery or other stores (OR: 1.14, 95% CI: 1.05–1.24, *p* < .05). Odds of overweight/obesity was higher among eligible non‐SNAP children who had greater access to convenience stores (OR: 1.11, 95% CI: 1.04–1.18, *p* < .05) Outcome 3: Alternative access measures of food exposure were not correlated with childhood overweight/obesity	8
Widome et al. (2009) USA	Cross‐sectional *N* = 4746 Age middle and high school students Gender: All	Food insecurity or food poverty: 2‐item adapted from the 1999 USDA Food Security/Hunger Core Module Weight status: Height and weight were measured by trained research staff and BMI was calculated Other variables: Household food availability was ascertained via 2‐scales	Outcome 1: Youths experiencing food insecurity were more likely to have a body mass index above the 95th percentile. Outcome 2: Compared with youths experiencing food security, youths with food insecurity had less food available in the home (both healthy (*p* = <.001) and unhealthy foods (*p* = <.001) Outcome 3: Youths experiencing food insecurity were less likely to eat family meals than those counterparts with a higher socioeconomic status (*p* = <.001) Outcome 4: Youths with food insecurity consumed more fast food but fewer breakfasts and family meals per week than those who experienced food security (Mean (95% CI): 2.03 (1.75, 2.31) *p* = .088)	8
Poulsen et al. (2019) USA	Cross‐sectional *N* = 817 Age 10–15 years youths and their adult parents Gender: All	Food insecurity or food poverty: The 6‐item USDA Food Security Scale Weight status: Trained staff measured height, weight, and waist circumference Other variables: Healthy and obesogenic home food availability (HFA) scales Self‐reported daily intakes of FV consumption by youths	Outcome 1: Compared with youth experiencing food security, youth from households with food insecurity had higher mean (beta (standard error)) BMIz (0.30 (0.15)) Outcome 2: Households with food insecurity had lower mean healthy HFA scores (−1.23 (0.54)); there was no evidence that obesogenic HFA differed between households by different food security status Outcome 3: Children from lower healthy HFA or higher obesogenic HFA families had fewer average daily intakes of FV (healthy HFA: 0.08 (0.02); obesogenic HFA: −0.06 (0.02)) Outcome 4: Consumption of fruits and vegetables was not associated with food security status. HFA did not appear to modify associations between food insecurity and weight outcomes	8
Matheson et al. (2002) USA	Cross‐sectional *N* = 124 Age 5th‐grade children (the average age of the children was 10.7 years; range from 9.9 to 12.5 years) and their mothers. Gender: All	Food insecurity or food poverty: The 18‐item US Department of Agriculture's Core Food Security Module Weight status: Weight and height were measured twice (BMI was then calculated). Other variables: Dietary intake (3 non‐consecutive 24‐h dietary recalls) Household food supplies were measured by a 40‐item household inventory	Outcome 1: The mean BMI for the sample was 20.70 ± 4.17 (range: 13.10–36.81). According to CDC growth curves, the average children's BMIs were at the 73rd percentile Outcome 2: Approximately 65.0% of households had food security, near 24.5% had food insecurity without hunger, almost 9% experienced food insecurity with hunger, and 1.6% were households experiencing food insecurity with severe hunger Outcome 3: Children from households experiencing food security were significantly heavier for their weight than were children from households experiencing food insecurity. The mean BMI for children from households with food security and food insecurity were 21.19 ± 4.69 and 19.80 ± 2.88, respectively (*p* = .04) Outcome 4: An inverse association between breads, cereals, and grains consumption at home and BMI was reported (*r* = −.21, *p* = .02). However, no significant associations were found between energy intakes and percentages of energy from fat and children's BMI Outcome 5: Food insecurity was adversely associated with the youths' BMIs and household food supplies but not with the youths' food intakes	6
Kral et al. (2017) USA	Cross‐sectional secondary analysis (was part of a larger laboratory‐based feeding study *N* = 50 Age mothers of 8–10‐year‐old children Gender: All	Food insecurity or food poverty: The 6‐item short form of the U.S. Household Food Security survey module Weight status: Children's and mother's weight were measured. Using the CDC Growth Charts, child age‐ and sex‐specific BMI percentiles and *z*‐scores were determined. Other variables: Child meal and snack patterns Child Feeding Questionnaire (CFQ): assessed how parents limited their child's access to food, and how parents pressured their children to eat more food Child Eating in the Absence of Hunger	Outcome 1: The odds of a child affected by obesity were 5 times higher for children from households with food insecurity compared with children from households experiencing food security (95% CI: 1.15–20.8) Outcome 2: 26% of families experienced food insecurity while the remaining families (74%) experienced food security Outcome 3: A greater percentage of children from households with food security ate 3–4 snacks per day (46 vs. 15.4%). However, a higher percentage of children from households experiencing food insecurity ate five or more snacks per day (15.4% vs. 0%) (*p* = .02) Outcome 4: Mothers from households with food insecurity reported greater concern about their child's weight and subsequently limited their child's access to food (*p* < .03) Outcome 5: Children from households with food insecurity indicated significantly greater external eating, both past satiation and in the absence of hunger (*p* < .03)	8
Watt et al. (2013) USA	Cross‐sectional *N* = 153 Gender: Females attending a clinic for prenatal care or for their child's 2, 6 or 12 months	Food insecurity or food poverty: 3‐item index on food security from the NHANES. Weight status: Weight was measured by clinic staff. Other variables: Mother's diet Infant's diet Data on participation in WIC and/or SNAP were obtained from the self‐administrated survey	Outcome 1: The prevalence of overweight/obesity was 11%. Over half of women (55%) had an infant profiling in the 85th percentile or higher Outcome 2: Majority of women did not meet dietary guidelines. Near 64% of women reported weekly eating of fast‐food. Daily drinking of SSB was prevalent among 44% of mothers. Limited access to food was common among three‐fourths of women Outcome 3: Most of the women breastfed after delivery. Near 25% of mothers reported feeding their 6‐ to 12‐month infant sweets on a weekly basis. Near 40% of mothers also reported they gave their infants high‐sugar fruit/vegetable juice daily Outcome 4: 64% of participants were WIC recipients and half of participants received food stamps (SNAP) over the past year Outcome 5: Maternal consumption of sweets and SSB were associated with infant overweight (*p* ≤ .05). Mothers who drink SSB daily were 4.7 times more expected to have a child in the 85th percentile or higher on weight for length (standard error: 0.673, *p* ≤ .05). Mothers who ate sweets twice a week or more were more than 11 times more expected to have a child in the 85th percentile or higher on weight for length (standard error: 0.892, *p* ≤ .05) Outcome 6: SNAP food stamp receipt was strongly correlated with risk of infant overweight (OR = 4.469, standard error: 0.693, *p* ≤ .05). Receipt of SNAP benefits was associated with significantly higher drinking of SSB (*p* ≤ .005). Mothers of SNAP receipt were near five times more likely to have a child profiling in the 85th percentile or higher	8
Smith and Richards (2008) USA	Cross‐sectional *N* = 202 Age youth 9–18 years, living in homeless shelters in Minneapolis, Minnesota Gender: All	Food insecurity or food poverty: A modified USDA's instrument on food security Weight status: Height and weight were measured. Other variables: Dietary intake: One single 24‐h recall was taken to collect information about type and quantity of food consumption, food preparation style, food distribution throughout the day, and where the food is eaten. Coping strategies to alleviate hungers	Outcome 1: 45% of boys and 50% of girls were at risk‐for‐overweight or were affected by overweight Outcome 2: More than 50% of children had no enough food in the house and 25% reported they went to bed hungry Outcome 3: Excessive servings of fats, oil, and sweet group were consumed by youths (18.6–22.7 servings) Outcome 4: Strategies to cope with food insecurity included overeating, eating anything, eating disliked foods, and eating at the homes of family and friends Outcome 5: Overeating was associated with eating at the home of family or friends and eating anything when really hungry (*p* < .05)	6

*Note*: JBI critical appraisal checklists, as appropriate to the study type, were used to assess the methodological quality of each included study. The scores indicate the reviewers (FE, KR) consideration of the possibility of bias in the design, conduct and analysis. Cross‐sectional studies, out of 8; Cohort studies, out of 11.

Reference: Joanna Briggs Institute (2017a).

Abbreviations: CI, confidence interval; FSP, Food Stamp Program; *SE*, standard error; SNAP, the Supplemental Nutrition Assistance Program; SSB, Sugar‐sweetened beverages; SSI, Supplemental Security Income; WIC, the Special Supplemental Nutrition Program for Women, Infants, and Children.

^a^ Scores for quality assessment (QA).

**TABLE 2:** Characteristics of the included studies and^a^ quality assessment; qualitative component

Author (date), country	Study type (size) & population	Phenomenon of interest	Key findings	QA score
Holston et al., (2020) USA	Focus groups *N* = 44	To explore experiences of food access and perceptions of food environments among low‐income people in the USA (mainly Black and female rural Louisiana residents) with high rates of obesity, poverty, and food insecurity	Outcome 1: Over half of participants reported running out of food before the end of the month. Almost all participants received SNAP benefits Outcome 2: Major themes were: store choice (price was most important deciding factor for most of participants), outshopping (having to leave the district to find lower prices and better quality foods), methods of acquiring foods other than the grocery store, and food insecurity (very few participants reported they go hungry, but indicated they sometimes struggle to feed their families) Outcome 3: Concerns regarding price, quality, and transportation were identified as factors negatively impacting food security. Participants perceived cost as a barrier to provide healthy foods for their families Participants reported they usually only had access to one grocery store in their districts, but they do not shop there because of perceived high food prices and poor food quality Outcome 4: In terms of potential solutions, providing job opportunities with reliable hours and a liveable wage were reported by participants as the major way to improve their food environment	7
Cooksey Stowers et al. (2020) USA	In‐depth, semi‐structured interviews with key stakeholders *N* = 10 Age: Adults	Key stakeholders' (such as food bank directors, food bank board members, advocate) perspectives on food banking system's role in the co‐occurrence risk of hunger and obesity disparities among clients using an equity‐oriented systems approach to examine the root causes of increased obesity risk among individuals with food insecurity	Outcome 1: Several themes indicated the link between obesity inequities and structural characteristics of the food banking system. These themes included (i) inadequate access to nutrient‐dense food resulted from access to unhealthy foods offered by donors, (ii) gaps in food supply vs. client needs; (iii) geographic disparity in access to health‐promoting resources; (iv) state‐level emergency food policy and programming; and (v) federal emergency food policy and programming Outcome 2: Themes that addressed the causes of ongoing risk of obesity inequality among individuals with very low food security were found to be (i) access to information; (ii) lack of inclusion/representation among leaders of food banks; (iii) mistrust in communities of color; and (iv) media representation and stereotypes about food pantry users Outcome 3: Many of stakeholders stated that improving the characteristics of the food banking system can alleviate obesity and hunger risks over time in the United States. However, two of them stated that other social issues such as poverty, racism and/or sexism as more important factors that should be addressed instead of improving the characteristics of food banking system alone as they believed that clients rely more on other types of food outlets than on pantries	9
Ong et al. (2021) Canada	Semi‐structured interviews *N* = 25 Age: Adults >18 years old	Relationships between food access, food security, health, and gentrification (the influx of higher income in‐movers) in a rapidly changing consumer food and nutrition environments	Outcome 1: The study revealed the complex decision‐making processes by residents to obtain healthy, affordable, and appropriate foods. It showed how they creatively cope with and adapt to changes within their food environments Outcome 3: Most participants expressed price as a main factor that affects their food destination choice (such as a mixture of supermarkets, discount stores, meal programs, food banks, charitable programs, and community kitchens) Outcome 4: To procure affordable food, some participants stated that they compromise food quality and personal morals	9
Byker Shanks et al. 2020 USA	Semi‐structured interviews *N* = 76 Mean age (*SD*) 40 (11.6)	Exploring food environments and diets among native American in the USA to inform programs, policy, and practice around food and nutrition	Outcome 1: Most participants self‐reported household prevalence of obesity (73.7%) Outcome 2: Food security, measured using 6‐item USDA HFSSM, was a major concern among participants with 50% of them reported low or very low food security status Outcome 3: Many participants highlighted participation in both federal nutrition assistance programs (51.9% participated in SNAP, and 27.8% used WIC, and etc) and community food assistance programs as important component of their community food environment. Some participants linked the history of such programs to their poor health outcomes (they stated a mistrust of modern food systems such as processing and large‐scale growing practices) Outcome 4: In terms of food access sites, many participants reported that groceries were mainly purchased, however, transportation was an issue for most of them because of long commute times and car sharing Outcome 5: Participants also reported reliance in food away from home due to time constraints	9
Gosliner and Shah (2020) USA	Focus group *N* = 55 (89% of participants were female and (89%) were between the ages of 18–50 years)	To capture the voices of those parents eligible for the Supplemental Nutrition Assistance Program Education (SNAP‐Ed) that aims to prevent obesity and chronic disease	Outcome 1: Most participants (62%) reported sometimes or often worrying about running out of food and 70% reported household participation in SNAP Outcome 2: Even those participants employed reported using SNAP, and/or charitable food assistance programs as they struggle to secure a consistent, healthy diet Outcome 3: Most participants stated that they want to eat healthfully, however, they find healthy eating too expensive (they described fruits and vegetables as unaffordable). In all groups, the most noticeable barrier to this was related to the challenge of inadequate income. They discussed about financial constraints because of inadequate income, high costs of housing, transportation, utilities, food, and other expenses. Outcome 4: Other key findings were related to the poor neighborhood food environments, and efforts to stretch food resources. Participants described relatively affordable unhealthy foods (junk foods at schools and corner stores, processed ready‐to‐heat microwaveable foods, etc.) as too easy to access. They stated that fast food restaurants were prevalent in their neighborhoods and their pricing strategies were reported to encourage unhealthy choices. They also noted about placement of cheap unhealthy foods in grocery stores as a barrier to healthy eating, especially those foods on sale. In fact, participants reported making purchases not based on their preferences but as tradeoffs between their food budgets and their food environment Outcome 5: In addition to a lack of money, participants reported to struggle with inadequate time. Low‐wage employment was associated with combined struggle of inadequate time and income. Participants stated that lack of time cause them to consume and feed their families junk food, fast food and highly processed prepared foods (such as microwaveable snacks) Outcome 6: Different food acquisition strategies were used by participants such as buying staple and shelf‐stable foods, buying foods on sale, searching food donations and food assistance benefits, and shopping at multiple stores to get the best deals	9
Jennings et al. (2020) USA	Community‐based participatory research approach *N* = 18 Indigenous children (8–12 years)	Children's food related perception of health and their beliefs about food insecurity and association with childhood obesity using photovoice (in a group of children who experienced high levels of food insecurity)	Outcome 1: Results indicated that urban Indigenous children experienced food and housing insecurity and they indicated unique holistic and socioecological perceptions about health Outcome 2: Children stated the following as healthy themes: nutrition, food sovereignty, gardening, interpersonal relationships, water quality, and natural and built environments Outcome 3: Food insecurity, access and cost, screen time, smoking, and violence were stated as unhealthy themes	9
Bhawra et al. (2015) Canada	Focus groups *N* = 32 parents and caregivers of metis and Off‐reserve first nations children	Factors influencing diets of children, families' experiences with food insecurity and coping strategies	Outcome 1: Low income was identified as a main cause of food insecurity and poor nutrition Outcome 2: A reliance on energy‐dense, nutrient‐poor foods was reported to be because these types of foods tend to be more affordable and last for longer time than more nutritious and fresh food options Outcome 3: A lack of transportation was reported to compromise families' ability to buy healthy foods Outcome 4: A lack of access to traditional foods were reported Outcome 5: Participation if food assistance programs such as food banks were not always reported as effective. They were sometimes reported to potentially exacerbate health problems, resulting in an increased rate of overweight and obesity	10
Franzen and Smith (2009) USA	Focus groups *N* = 65 adults	To understand how food insecurity and environmental factors influence dietary behavior, BMI, and health	Environmental changes negatively associated with weight and health of Hmong adults	10
Genuis et al. (2015) Canada	Photovoice investigation *N* = 26 young children	To deeper understand food security and to explore youths' experiences and perceptions of food. Children took photographs of the food they consumed	Outcome 1: Children reported a dualistic understanding of healthy against unhealthy foods Outcome 2: Dominant foods were packaged and quick‐preparation foods Outcome 3: A Small Number Of traditional foods were shown in the photographs. However, these foods were viewed as central to Aboriginal health Outcome 4: Even though smaller numbers of fruits and vegetables were depicted in photos, children liked to consume these foods when they were available at their home	10
Kerpan et al. (2015) Canada	Focus groups – Ethnographic study *N* = 15 students in grades 9	Determinants of diet among urban aboriginal youths	Outcome 1: Traditional foods were reported to be healthy and liked by participants Outcome 2: The daily challenges the participants faced with food insecurity were shown in the theme “The Struggle”	10
Thompsonet al. (2018) UK	Ethnographic study *N* = 42 participants (this was a mixed sample in terms of gender, age, ethnicity, and immigration status)	Health and wellbeing challenges related to food poverty and food banking in London	Food poverty was believed to be a barrier to providing adequate care and nutrition for young children. Lack of access to adequate fresh food, food storage and cooking facilities were reported to exacerbate food poverty. it was believed that food poverty intensifies existing health and social problems	10

*Note*: JBI critical appraisal checklists, as appropriate to the study type, were used to assess the methodological quality of each included record. The scores indicate the reviewers (FE, KR) consideration of the possibility of bias in the design, conduct and analysis. Qualitative studies, out of 10.

Reference: Joanna Briggs Institute (2017b).

^a^ Scores for quality assessment (QA).

### 3.2 Methodological quality

Quantitative studies: Thirty‐five cross‐sectional studies were included in this review. Overall quality scores ranged from six to eight out of 8 (Table 1 and Table S3). One longitudinal study was also included, having a maximum overall quality score of 11 (Table 1 and Table S4). Thus, the studies were deemed to be of very good quality, with the risk of selection bias remaining low.

Qualitative studies: Eleven studies were critically appraised and considered of very good quality (Table 2 and Table S5). Overall quality scores for these studies ranged from 7 to 10 out of 10, indicating a high level of data integrity and congruity between methodology and the research aims, data collecting methods, and analysis.

#### 3.2.1 Characteristics of food insecurity exposures

Tools included in studies for assessment of food insecurity status were 6‐item US Household Food Security Survey Module (HHFSM) (Bauer et al., 2012; Bruening et al., 2012; Hooper et al., 2020; Kaiser et al., 2019; Kral et al., 2017; Leung & Villamor, 2011; Poulsen et al., 2019; Ro & Osborn, 2018; Shinwell et al., 2021). 18‐item the Food Security Core Module (FSCM) or simply the US Household Food Security Survey Module (USDA HFSSM) (i.e., by ‘simply’ we mean that FSCM and USDA HFSSM are the same tool, but are called either interchangeably; Benjamin‐Neelon et al., 2020; Domingo et al., 2021; Matheson et al., 2002; McCurdy et al., 2015; Mercille et al., 2012; Robaina & Martin, 2013; Rodriguez et al., 2021; Santarossa et al., 2021; van der Velde et al., 2020; Vedovato et al., 2016; Webb et al., 2008), 10‐item US adult food security survey (Keenan et al., 2021; Sanjeevi et al., 2018; Walch & Holland, 2021; Webb et al., 2008; Yau et al., 2020), the National Health and Nutrition Examination Survey Food Security Survey Module (NHANES FSSM; Nguyen et al., 2015; Watt et al., 2013), 10‐item Radimer/Cornell Hunger Scale (Dharod et al., 2013), 11‐item USDA Adult Food Security Survey Module (Huelskamp et al., 2021), USDA's 30‐day Adult Food Security Scale (Gorski Findling et al., 2018), 2‐item adapted from the 1999 USDA Food Security/Hunger Core Module (Widome et al., 2009), the Hunger Vital Sign (HVS) screener tool (Wirth et al., 2020), and a modified USDA's instrument on food security (Smith & Richards, 2008; Vadiveloo et al., 2020).

#### 3.2.2 Type of food environment characteristics

In this review, food environments were defined as objective (e.g., geographic information system) and/or perceived aspects of the physical and economic food environment inside and outside the home. Included studies were diverse in their measures and in their results for the food environment component.

##### 3.2.2.1 Home food environment

Two studies used home food availability (HFA) scales; in a cross‐sectional study of 817 individuals, healthy and obesogenic HFA scales were used to assess how frequently particularly foods were available at the home (Poulsen et al., 2019). In another study of 432 parents or caregivers of kindergarten‐age children, HFA was assessed using yes or no questions asking about availability of different types of fruits and vegetables (FV), energy‐dense foods, and beverages in their homes (Bauer et al., 2012).

In a study of 4589 middle and high school students, household food availability was ascertained via two scales (Widome et al., 2009), measuring both the availability of healthy and unhealthy foods in their homes. Fast food intake was also determined by asking how often they ate something from a fast‐food restaurant during past week.

In a study of 2095 parents, participants reported on home food environment using different items. Four items assessed perceptions of access to fruit and vegetables, addressing quality, variety, and cost of produce (Bruening et al., 2012). The types of food consumed at family meals were measured using six items and one item reported on family meal frequency. Fast‐food consumption was also assessed.

A cross‐sectional study of 152 females participating in Supplemental Nutrition Assistance Program (SNAP) program (aged 18–50 years) used a multi‐dimensional home environmental scale (MHES). This scale was created to measure home environment from the perspective of adolescent children and their mothers (Sanjeevi et al., 2018). The environmental influence was measured by questions related to availability of specific healthy and unhealthy food items at home (Sanjeevi et al., 2018).

In a study of 124 largely Hispanic and fifth‐grade children (aged 9–13 years), their mothers provided reports of household food supplies (Matheson et al., 2002). Mothers completed a 40‐item household inventory of food supplies.

In a cross‐sectional secondary study, 50 mothers of 8‐ to 10‐year‐old children completed different questionnaires (Kral et al., 2017). Using these, meal and snack patterns of children, restriction of child's access to food by parents, extent to which children were pressured to eat more food, and children's susceptibility for eating more in the presence of palatable foods were assessed.

##### 3.2.2.2 Neighborhood fresh produce environment

Two measures were included in one study (18–65 years old; Ro & Osborn, 2018) that measured perceptions of healthy foods in the neighborhood food environment, including availability and affordability of FV. For availability, participants answered how often they found fresh FV in their neighborhood. For affordability of FV, they answered if the fresh FV in their neighborhood were affordable. In a study of 301 individuals who resided within communities with food security and food insecurity, respondents answered about household's perception about different food accessibility within their neighborhood. They answered questions about easy access to fresh FV, and food support services such as food pantry (Kaiser et al., 2019).

##### 3.2.2.3 Food source destinations (neighborhood food access)

Twelve studies examined different types of neighborhood food access. In a large cross‐sectional study of 3748 children (2–18 years old), neighborhood retail food access was measured using numbers of food outlets such as stores, and restaurants located within 1 mile of youths' home (Gorski Findling et al., 2018). Access was reported for specific food outlet types such as fast‐food and non‐fast‐food restaurants, convenience stores, supermarkets, grocery stores, and other stores. Alternative neighborhood food access measures included store type of closest the SNAP retailer (Gorski Findling et al., 2018). Household food purchases and acquisitions were evaluated. To measure spending on unhealthy food items, the mean of sugary beverage spending by each child's household was also examined (Gorski Findling et al., 2018). Another cross‐sectional survey of adults (*n* = 298) measured the sources of foods that were purchased from over the past month (Vedovato et al., 2016). The food sources included fast‐food restaurants, convenience stores, bar or pub, food pantry, family and friends, church or community centre, street food vendor, etc. (Vedovato et al., 2016). A study of parents or caregivers of kindergarten‐aged children asked parents to report the frequency of family fast‐food visits per week (*n* = 432; Bauer et al., 2012).

In a study of adults aged 18 years and over who lived on low‐income neighborhoods (*n* = 435), participants were asked to answer questions regarding use of supermarkets or other store types for food purchases and use of free or low‐cost food from charitable sources (i.e., food banks, soup kitchens, church or community outreach programs, shelters, friends, and/or family; Webb et al., 2008). A study of 212 food pantry users asked the participants about frequency of going to food pantries, and/or to soup kitchens (Robaina & Martin, 2013). Participants used food pantries on a long‐term basis, with 62.5% visiting at least once per week and 44% ate foods at a soup kitchen. A correlational study of 166 mothers of 2–5‐year‐old children on a low income used 8‐item Food Shopping Practices scale to ask about the use of shopping habits to stretch food dollars (i.e., to rate their use of food coupons, buying lower cost food to save money, shopping at specific stores due to a sale, buying food in bulk, and using a shopping list) in the previous 30 days (McCurdy et al., 2015). Respondents also answered questions about using emergency food parcels from soup kitchens, food banks, community cupboards, or churches (McCurdy et al., 2015).

In another study of 153 women on a low income, a self‐administrated survey asked women about their perceived food access (Watt et al., 2013). Seventy‐five percent of women reported that they experienced limited food access. Finally, a cross‐sectional study of 107 women responsible for household food supplies measured grocery shopping practices and access to traditional foods (Mercille et al., 2012). Most families made their grocery shopping from a supermarket within 145 km or more from their homes.

##### 3.2.2.4 Food source destinations –nutrition assistance programs

Nine studies assessed participation in nutrition assistance programs; the interactions between SNAP participation, food insecurity, and BMI were examined in a study of 2003–2010 US NHANES (*n* = 8333; Nguyen et al., 2015). SNAP is previously recognized as food stamps and is the largest federal program in the United States that offers support for the purchase of foods to low‐income US households to alleviate food insecurity (Sachdev et al., 2019). In a cross‐sectional study of 7741 adult California Health Interview Survey, SNAP participation was assessed using questions related to receiving food stamp benefits. Supplemental Security Income (SSI) participation was also assessed (Leung & Villamor, 2011). In a cross‐sectional analysis of 3748 children, household participation in SNAP and WIC was examined (Gorski Findling et al., 2018). In a study of 435 adult residents of low‐income neighborhoods, participation in 3 government nutrition assistance programs (i.e., the free or reduced‐price school meals program, the Food Stamp Program [FSP]), and the Special Supplemental Nutrition Program for Women, Infants, and Children [WIC]) was assessed (Webb et al., 2008). In a study of 212 food pantry users, participants were asked whether they receive SNAP or WIC (Robaina & Martin, 2013). Over half (57%) of participants received SNAP. A cross‐sectional, correlational study of 166 mothers of young children on a low income assessed participation in government food assistance programs. Participants answered questions from a modified version of the Current Population Survey Food Security Supplement (FSS) to assess participation in SNAP and WIC (McCurdy et al., 2015). Eighty percent of participants reported receipt of benefits from SNAP. In a study of 153 women on a low income, data on participation in WIC and/or SNAP were obtained from the self‐administrated survey (Watt et al., 2013). Sixty‐four percent of participants used the benefits obtained from WIC and half of them benefited from food stamps (SNAP) over the previous year.

##### 3.2.2.5 Healthy food beliefs and attitudes

Five studies measured this aspect of food environments; in a cross‐sectional survey of 298 adults, four subscales were developed to indicate different aspects of beliefs and opinions about healthy foods: affordability, convenience, importance, and taste (Vedovato et al., 2016). In a study of 432 parents or caregivers of kindergarten‐aged children, barriers to healthy foods at the home were assessed (Bauer et al., 2012). A cross‐sectional study of 107 females who were responsible for household food supplies measured self‐efficacy for food preparation using the calculation of two self‐efficacy scores. One scale measured food preparation in general and another one measured healthy food preparation (Mercille et al., 2012). Self‐efficacy in food preparation was described as individuals' confidence in their ability to make dishes and balanced meals using store‐bought food. Women were fairly confident about their capability to prepare store‐bought food. However, the average score for self‐efficacy in healthy food was slightly lower, suggesting more difficulty in this regard (Mercille et al., 2012). They also reported on their perceptions of FV supply in local stores. The local grocery store was perceived negatively, as it did not usually bring fresh FV and it was used mainly as a backup.

##### 3.2.2.6 Dietary intake (diet quality)

This determinant was reported as intake of specific foods or food groups are associated with obesity and such diets may relate to different aspects of neighborhood food environments.

Thirteen studies measured dietary intakes of participants; in a cross‐sectional analysis of 7741 Adult California Health Interview Survey, dietary information was collected by asking about the frequency of eating of fruits (excluding fruit juice); vegetables (excluding fried potatoes); soda (excluding diet soda); French fries, and fast food (Leung & Villamor, 2011). A study of 432 parents or caregivers of kindergarten‐aged children, parents were asked about the frequency of fast‐food visits per week. They were also asked about the frequency of their child food consumption from hot or ready‐made food from a convenience store or gas station over the past 30 days (Bauer et al., 2012). Another study of 212 food pantry users, diet quality of participants was measured using the Block Food Frequency Screener. These users reported about their usual consumption of fruit, vegetables, and fiber (Robaina & Martin, 2013). A cross‐sectional study of 202 young people (9–18 years) who were homeless and were living in two of the largest family shelters in the USA assessed dietary intake by completing a single 24‐h recall to provide information about the type and quantity of food consumed, preparation style, where food is eaten, and how it is spread over the day (Smith & Richards, 2008). Dairy, fruits, and vegetables were consumed less than recommended levels (below the estimated average requirements) by both males and females of all ages. All youths ate excessive servings of sweet groups, fats, and oil (18.6–22.7 servings). Another cross‐sectional study of 195 Somali refugee women in the United States estimated their regular dietary intakes by completing a short food frequency questionnaire. Questions were asked to estimate how often specific food items were consumed such as eggs, meats, beans/lentils, grains, dairy, fruits, and vegetables (Dharod et al., 2013). In a study of 153 women on a low income, mother's diet was measured using an 8‐item index from Starting the Conversation (Watt et al., 2013). It included questions about intakes of FV, sugar‐sweetened beverages (SSB), high‐fat foods, and desserts. Infant's diet was evaluated as breastfeeding initiation and consumption of particular foods such as fruits and French fries. The majority of women did not meet dietary guidelines and nearly 64% of them reported weekly intakes of fast‐food. Drinking SSB at a daily basis was reported by 44% of women. Most of the women breastfed after delivery. Usual feeding practices were that 39% of women reported they gave their infants high‐sugar fruit/vegetable juice daily and 24% of them reported feeding their infants sweets on a weekly basis (Watt et al., 2013).

##### 3.2.2.7 Coping strategies to alleviate hunger

One cross‐sectional study of 202 young people (9–18 years) evaluated coping strategies used by youths who were homeless and were living in two of the largest family shelters in the USA (Smith & Richards, 2008). Coping strategies to alleviate food insecurity included overeating, eating at the homes of family and friends, eating disliked foods, and eating anything.

### 3.3 Findings of the review

#### 3.3.1 Quantitative component

##### 3.3.1.1 Associations between food insecurity and BMI

**FIGURE 2:**
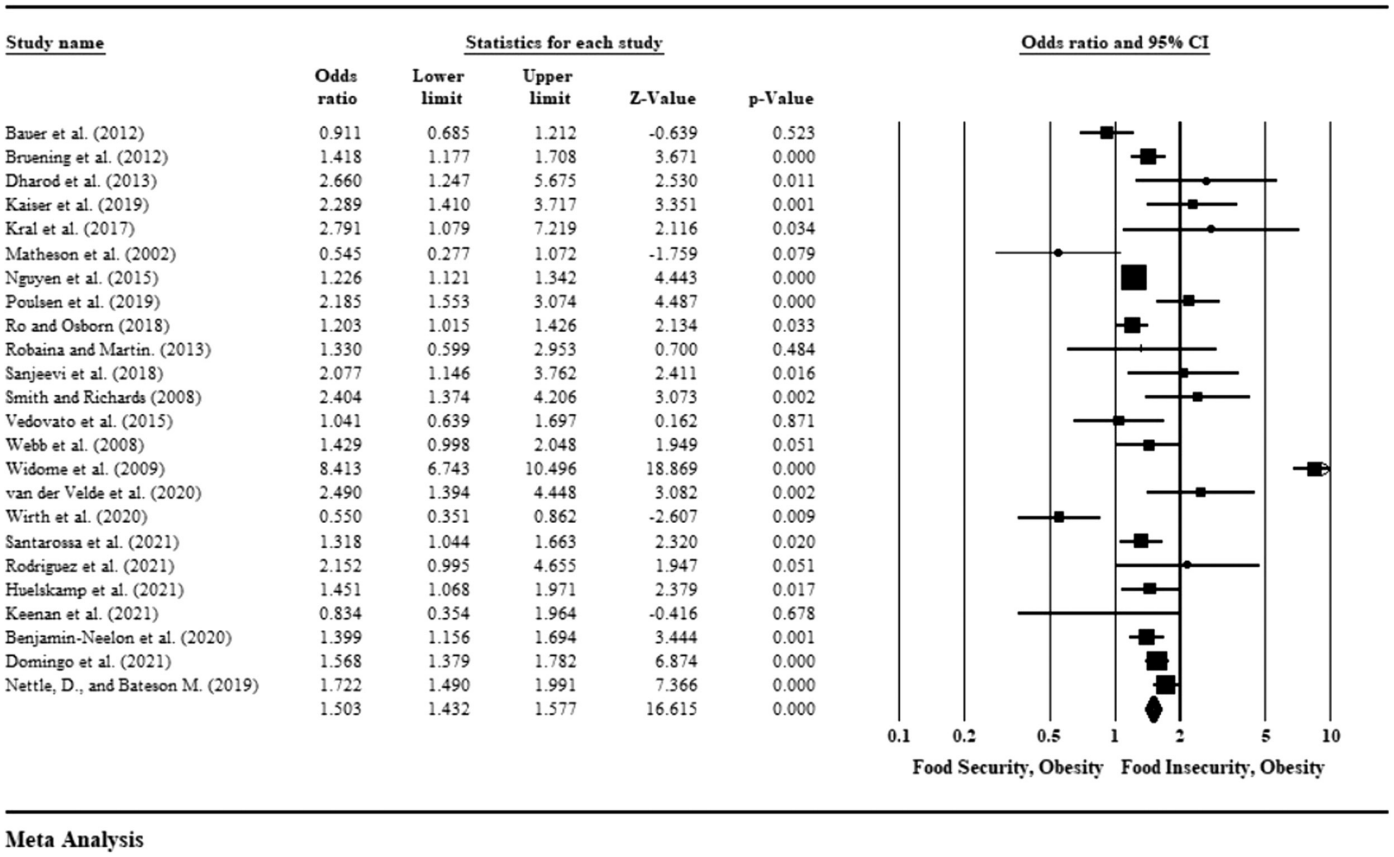
Forest plot showing OR with 95% CI of the association between food security status and obesity.

To analyze the association between food insecurity and obesity, the OR of 36,113 cases in 24 studies (Bauer et al., 2012; Benjamin‐Neelon et al., 2020; Bruening et al., 2012; Dharod et al., 2013; Domingo et al., 2021; Huelskamp et al., 2021; Kaiser et al., 2019; Keenan et al., 2021; Kral et al., 2017; Matheson et al., 2002; Nettle & Bateson, 2019; Nguyen et al., 2015; Poulsen et al., 2019; Ro & Osborn, 2018; Robaina & Martin, 2013; Rodriguez et al., 2021; Sanjeevi et al., 2018; Santarossa et al., 2021; Smith & Richards, 2008; van der Velde et al., 2020; Vedovato et al., 2016; Webb et al., 2008; Widome et al., 2009; Wirth et al., 2020), involving both adults and children, was pooled together for the meta‐analysis. These studies used a cross‐sectional approach in addition to one cohort study. As shown in Figure 2, meta‐analysis of these studies showed an overall small but statistically significant association between food insecurity and obesity (OR: 1.503, 95% CI: 1.432–1.577, *p*‐value = .000) when all ORs were combined with the random‐effects model. This means food insecurity increased the risk of obesity among adults and children. Therefore, individuals experiencing food insecurity were more likely to be affected by obesity.

##### 3.3.1.2 Associations between food environments and BMI

###### 3.3.1.2.1 Home food environment

A cross‐sectional study of 10‐ to 15‐year‐old youths and their parents (*n* = 817) reported no associations between obesogenic HFA scores and body mass index *z* score (BMIz) (beta coefficient: −0.02 [95% CI: −0.04–0.01, *p* = .143]; Poulsen et al., 2019). In this study, there was no evidence that healthy or obesogenic HFA changed the food security or anthropometric associations in children. This study also found that households living with food insecurity had significantly lower mean healthy HFA scores compared to households living in food security (beta coefficient: −1.23 [95% CI: −2.29 to 0.18, *p* = .022]). But there was no evidence of a difference in mean obesogenic HFA scores (beta coefficient: −1.02 [95% CI: −2.32 to 0.28, *p* = .122]). In this study, young people from higher obesogenic HFA or lower healthy HFA households had fewer mean daily FV intakes (beta for healthy HFA: 0.08 (95% CI: 0.03–0.12, *p* = .001); beta for obesogenic HFA: −0.06 (95% CI: −0.09 to −0.02, *p* = .003).

Another study of parents or caregivers of kindergarten‐aged children (*n* = 432) found no differences in families' HFA by food security status (Bauer et al., 2012).

A study of middle and high school students (*n* = 4589) demonstrated that youths experiencing food insecurity had several eating‐related risk factors for overweight/obesity (Widome et al., 2009). Adolescents who “often” did not have enough to consume or that they suffered from hunger “some months” reported eating more fast food than did those who had food security (Widome et al., 2009; mean score [95% CI]: 2.03 [1.75, 2.31] *p* = .088). This group had the greater percentage of young people who were affected by obesity (≥95th percentile) (*p* = .01). These youths had less food available in the home (both healthy [*p* < .001] and unhealthy foods [*p* < .001]). This study found that youths with food insecurity were less likely to eat family meals than peers of a higher socioeconomic status (*p* < .001). The study suggested that this might be because of limited or irregular food availability that might be less likely to establish a regular family meal routine (Widome et al., 2009).

The population‐based study of 2095 parents showed that the home food environment in households living with food insecurity was poorer than in households living in food security (Bruening et al., 2012). Parents experiencing food insecurity reported having more fast food at family meals (95% CI = 0.1, 0.2, *p* < .01) and more serving of SSB (95% CI = 2.5, 10.3, *p* < .01) at family meals than parents living in food security. They also reported serving frequently less healthy food items such as green salad, vegetables, and fruits (*p* < .05). The study explained that the higher BMI and poorer eating patterns of parents with food insecurity may be due to the fact that more obstacles in accessing healthy foods such as FV were reported by this group than parents experiencing food security. Great differences in perceived access to FV were reported between parents living in food security and parents living with food insecurity. For example, near 40% of parents experiencing food insecurity compared to near 14% of parents experiencing food security perceived that fruits were too expensive to purchase (95% CI = 21.5, 30.6, *p* < .01). Parents affected by food insecurity were 3–4 times more likely to find FV to be too expensive. These parents believed that the quality and variety of available FV were poor (*p* < .01).

Another study of 152 participants in SNAP program, availability of unhealthy food was significantly associated with BMI (beta coefficient = −0.227, *p*‐value = .02; Sanjeevi et al., 2018). Also, a significant difference between groups living in food security and groups living with food insecurity was reported for availability of unhealthy foods at home. Groups experiencing food security scored higher than groups with food insecurity for the availability of unhealthy foods at home by almost 14% (*p* < .01). This study indicated that the relationship between food insecurity and obesity was partially mediated by home food environment (beta coefficient: 0.19, 95% CI: 0.01–0.42, *p* < .05). Thus, home food environment could play a vital role in mediating this relationship in this population.

The study of Hispanic and fifth‐grade children (*n* = 124) demonstrated a significant association between household food supplies and household food security (*p* < .01). Household food supplies were significantly correlated with youth's consumption of fruit, meat, sweets, and snacks at home (*p* < .05; Matheson et al., 2002).

A cross‐sectional secondary study of 50 mothers of 8‐ to 10‐year‐old children (*n* = 100) found large percentage of youths from households living in food security reported eating 3–4 snacks per day (46% vs. 15.4%), while a greater percentage of youths from households living with food insecurity reported eating 5 or more snacks per day (15.4% vs. 0%) (*p* = .02; Kral et al., 2017). Mothers from households experiencing food insecurity reported significantly more concern regarding their child's weight and subsequently limited access to food by their children at a greater extent than mothers from households living in food security (*p* < .03). Children from households with food insecurity had significantly more external eating, both past satiety and in the absence of hunger (*p* < .03).

###### 3.3.1.2.2 Neighborhood fresh produce environment

In a cross‐sectional study of 5957 individuals, the OR between overweight/obesity and food insecurity became nonsignificant when the perceptions of neighborhood fresh produce environment were added (OR = 1.32, 95% CI: 0.89–1.98; Ro & Osborn, 2018). Only neighborhood affordability of fresh produce was statistically associated with overweight/obesity; women affected by severe food insecurity (in this study, there was no difference in the percent of overweight/obesity based on the food insecurity status for men) had lower odds of obesity when they usually or always afforded fresh FV in their neighborhood (OR = 0.70, 95% CI: 0.53–0.93; Ro & Osborn, 2018). This suggested that neighborhood affordability of fresh produce determined the statistical correlation between food insecurity and overweight/obesity.

A study of people who lived within food secure and food insecure communities (*n* = 301) demonstrated that those who lived in the food secure areas had the greatest satisfaction with easy access to FV. Food assistance users had higher incidences of obesity (OR: 1.46, 95% CI: 0.54–3.94) (the overall *p*‐value, however, was not significant). Perceived farmers' market access was associated with a lower prevalence of overweight/obesity (OR: 0.46, 95% CI: 0.17–1.23) (the overall *p*‐value, however, was not significant; Kaiser et al., 2019).

###### 3.3.1.2.3 Food source destinations (neighborhood food access)

A study of 2–18 years old children (*n* = 3748) demonstrated that more neighborhood access to combination groceries or other types of stores was related to greater prevalence of obesity among children overall and those participated in SNAP (Gorski Findling et al., 2018). Odds of childhood overweight/obesity were higher with greater access to combination grocery/other stores overall (OR: 1.10, 95% CI: 1.03–1.17, *p* < .05) and for children in SNAP (OR: 1.14, 95% CI: 1.05–1.24, *p* < .05; Gorski Findling et al., 2018). In this study, alternative access measures of food exposure were not associated with child overweight/obesity (Gorski Findling et al., 2018). The average child lived in a household in which 6.3% of their total spending at food outlets was on sugary beverages (Gorski Findling et al., 2018). Compared to non‐SNAP households, the average youths from households participated in SNAP also spent a higher percentage of their budget on sugary beverages (*p* < .05; Gorski Findling et al., 2018).

A cross‐sectional survey of 298 households found no significant associations between food source use patterns (such as shopping from a convenience or grocery store) and excess body weight (Vedovato et al., 2016).

A study of 432 parents and caregivers of kindergarten‐aged children found no differences in frequency of families' fast‐food visits by food security status (Bauer et al., 2012).

In a study of 435 adults who were residents of low‐income neighborhoods, BMI was significantly higher for those cases who acquired their foods from charitable sources such as food banks or soup kitchens (*p* < .01). Also, those who reported shopping at convenience stores (*p* = .04) and those who consumed fast‐foods in the month prior to the survey had significantly higher BMI (*p* < .01; Webb et al., 2008). In this study, no association was found between BMI and different use of supermarkets, ethnic grocery stores, or use of farmers' markets.

A study of mothers of young children on a low income (*n* = 166) found no significant association between maternal BMI and number of weekly shopping visits to supermarkets (McCurdy et al., 2015). However, maternal BMI was significantly associated with variables related to food resources. Use of community food programs (*p* < .05) and more frequent use of food shopping practices to stretch food dollars (*p* = .04) were positively associated with maternal BMI.

In a study of women who were responsible for household food supplies (*n* = 107), no association was found between self‐efficacy scores, grocery shopping practices, and access to traditional food (Mercille et al., 2012).

###### 3.3.1.2.4 Food source destinations –nutrition assistance programs

Analysis of NHANES 2003–2010 (*n* = 8333) showed that, while those experiencing food insecurity or SNAP participants had a higher BMI and greater possibility of obesity (*p* < .05), the combined association of food insecurity and SNAP participation indicated a decrease in BMI across all three groups of food insecurity (*p* < .05) and reduced the chance of obesity among those who had marginal food security (*p* < .05; Nguyen et al., 2015).

A cross‐sectional analysis of 7741 Adult California Health Interview Survey demonstrated that the incidence of obesity was 30% higher among those who participated in SNAP than among the nonparticipants (*p* = .01; Leung & Villamor, 2011). This association was more evident among males than females. Participation in SSI programs was positively associated to an adjusted 50% higher incidence of obesity compared to those who did not participate.

A large cross‐sectional study of 3748 children demonstrated that youths from SNAP families had higher odds of overweight/obesity with greater access to combination grocery/other stores (OR: 1.14, 95% CI: 1.05–1.24, *p* < .05). Eligible non‐SNAP youths had higher odds of overweight/obesity with greater access to convenience stores (OR: 1.11, 95% CI: 1.04–1.18, *p* < .05; Gorski Findling et al., 2018).

In a study of 435 adult residents of low‐income neighborhoods, compared to those who reported no federal nutrition assistance, those who participated in the WIC, FSP, and free/reduced‐price school meals during the 12 months before to the survey had significantly higher BMI (*p* < .01; Webb et al., 2008). However, participation in FSP on a chronic basis (≥6 months) was linked to lower BMI compared to those who participated for <6 months (*p* < .01; Webb et al., 2008).

A cross‐sectional, correlational study of 166 mothers of young children on a low income reported that participation in WIC or SNAP did not appear to be as considerable determinants of maternal BMI (McCurdy et al., 2015). In a study of 153 women on a low income, risk of overweight in infants was strongly associated with participation in the SNAP FSP (OR = 4.469, standard error: 0.0693, *p* ≤ .05; Watt et al., 2013). Participation in SNAP was significantly associated with greater intakes of SSB (*p* ≤ .005). Those women participated in SNAP were 4.5 times more likely to have a child in the 85th percentile or higher. SNAP participation contributed to child obesity through increased mothers' intakes of SSB.

###### 3.3.1.2.5 Healthy food beliefs and attitudes

A cross‐sectional survey of 298 households demonstrated that those who greatly perceived healthy food as being convenient had a 57% decline in odds of BMI for caregivers and children (OR: 0.43, 95% CI: 0.21–2.4, *p* < .05; Vedovato et al., 2016). This study found that compared to those groups who had food insecurity, those participants who had food security more reported to agree that healthy foods are affordable and convenient. After adjusting for socioeconomic characteristics, odds of household food insecurity were 0.18 (95% CI: 0.09, 0.39) and 0.49 (95% CI: 0.24, 0.95) among those families who perceived healthy food to be affordable and convenient, respectively (*p* < .05; Vedovato et al., 2016).

A study of 432 parents or caregivers of kindergarten‐aged children found that food security status changed parents' experience of barriers to having healthful food in their homes (Bauer et al., 2012). Parents experiencing food insecurity were most likely to report that there was little variety of FV where they buy groceries (*p* = .003) and were more likely to agree that where they buy groceries the fruits and vegetables were in poor condition (*p* = .03). These parents were also more likely to report that their family does not like FV (*p* = .01).

A cross‐sectional study of 107 women responsible for household food supplies demonstrated among BMI categories, only women with severe obesity had less confidence in their healthy food preparation abilities (beta coefficient: −0.23, *p* = .03; Mercille et al., 2012). Both self‐efficacy scores were inversely associated with severe household food insecurity (beta coefficient: −0.25, *p* = .01); however, the association became nonsignificant for the general food preparation score. Lack of availability as an excuse for not buying FV locally was positively correlated with self‐efficacy in healthy food preparation (beta coefficient: 0.29, *p* < .01 (Mercille et al., 2012).

###### 3.3.1.2.6 Dietary intake (diet quality)

Thirteen studies measured dietary intakes of participants. A cross‐sectional analysis of 7741 adults in the California Health Interview Survey demonstrated that SNAP and SSI participants had higher consumption of soda than nonparticipants of any program (Leung & Villamor, 2011). The findings from another study of 432 parents or caregivers of kindergarten‐aged children demonstrated that children from families who experienced very low food security had higher intakes of hot or ready‐made foods bought from a convenience store or gas station than those youths from families living in food security (*p* = .002). Children living with food insecurity also ate pizza and fried chicken more often than children living in food security (*p* < .05; Bauer et al., 2012). Based on the results from a study of 212 food pantry users, participants living in food security were twice as likely to eat fruit, vegetables, and fiber than those who had food insecurity (OR = 2.3, 95% CI: 1.1, 5.2, *p* = .05; Robaina & Martin, 2013).

A cross‐sectional study of 202 young people who were homeless found significant associations with overeating (as a coping strategy) and higher food intakes of fat (*p* = .037), protein (*p* = .010), and the meat food group (*p* = .014) among females 9–13 years (Smith & Richards, 2008). Among youths' males 9–13 years, overeating was associated with increased intakes of calories (*p* = .012), carbohydrates (*p* = .030), fat (*p* = .011), protein (*p* = .015), bread (*p* = .028), and vegetables (*p* = .029). Eating at the homes of family and friends, as the coping strategies, was also associated with overeating (*p* = .017; Smith & Richards, 2008). These results suggested that these youths have used coping strategies for dealing with a food insecure environment by overeating and consuming high‐fat foods when tasty food was available. In this study, the major calorific snacks that were identified to be commonly consumed by youth were salty snacks, candy, soft drinks, fruit drinks, French fries, cheeseburgers, and pizza. These types of foods are largely offered by fast‐food restaurants and convenience stores, which are common stores in downtown urban neighborhoods (Smith & Richards, 2008). Another cross‐sectional study of 195 Somali refugee women in the United States demonstrated an association between BMI scores and daily intake from different food groups. A significant difference was noted in the fruits, vegetables, and beans groups (Dharod et al., 2013). Intake from all of these food groups at least once a day was less common among participants who were affected by overweight/obesity than individuals with normal weight (*p* ≤ .05). Families with severe level of food insecurity or child hunger had higher intakes of eggs. Child hunger was 20 times greater among households who consumed eggs at least once a day (OR: 21.20; 95% CI: 7.83–57.34; *p* < .001). Daily intake of FV also predicted food security. When participants reported eating leafy green vegetables at least once a day, the odds of food insecurity became 70%–80% lower (OR: 0.02; CI: 0.08–0.51; *p* < .001; Dharod et al., 2013). Finally, in a study of 153 women on a low income, while mothers had possible risk factors for childhood obesity such as fast‐food consumption, intakes of sweets and SSB by mothers were associated with infant overweight (*p* ≤ .05; Watt et al., 2013). Mothers who drink SSB daily were 4.7 times more expected to have a child in the 85th percentile or higher on weight for length (standard error: 0.673, *p* ≤ .05). Mothers who ate sweets twice a week or more were more than 11 times more expected to have a child in the 85th percentile or higher on weight for length (standard error: 0.892, *p* ≤ .05).

###### 3.3.1.2.7 Coping strategies to alleviate hunger

In a cross‐sectional study of 202 young people who were homeless, regression analyses of coping mechanisms found that the variable “when I am really hungry, I will eat anything” was predictive of males' BMI (beta coefficient: −1.046 [standard error: 0.426, *p* = .000]). For females, the variable “if I am hungry, I will eat foods that I do not like” was predictive of BMI (beta coefficient: −0.804 [standard error: 0.379, *p* = .036]).

#### 3.3.2 Qualitative component

**FIGURE 3:**
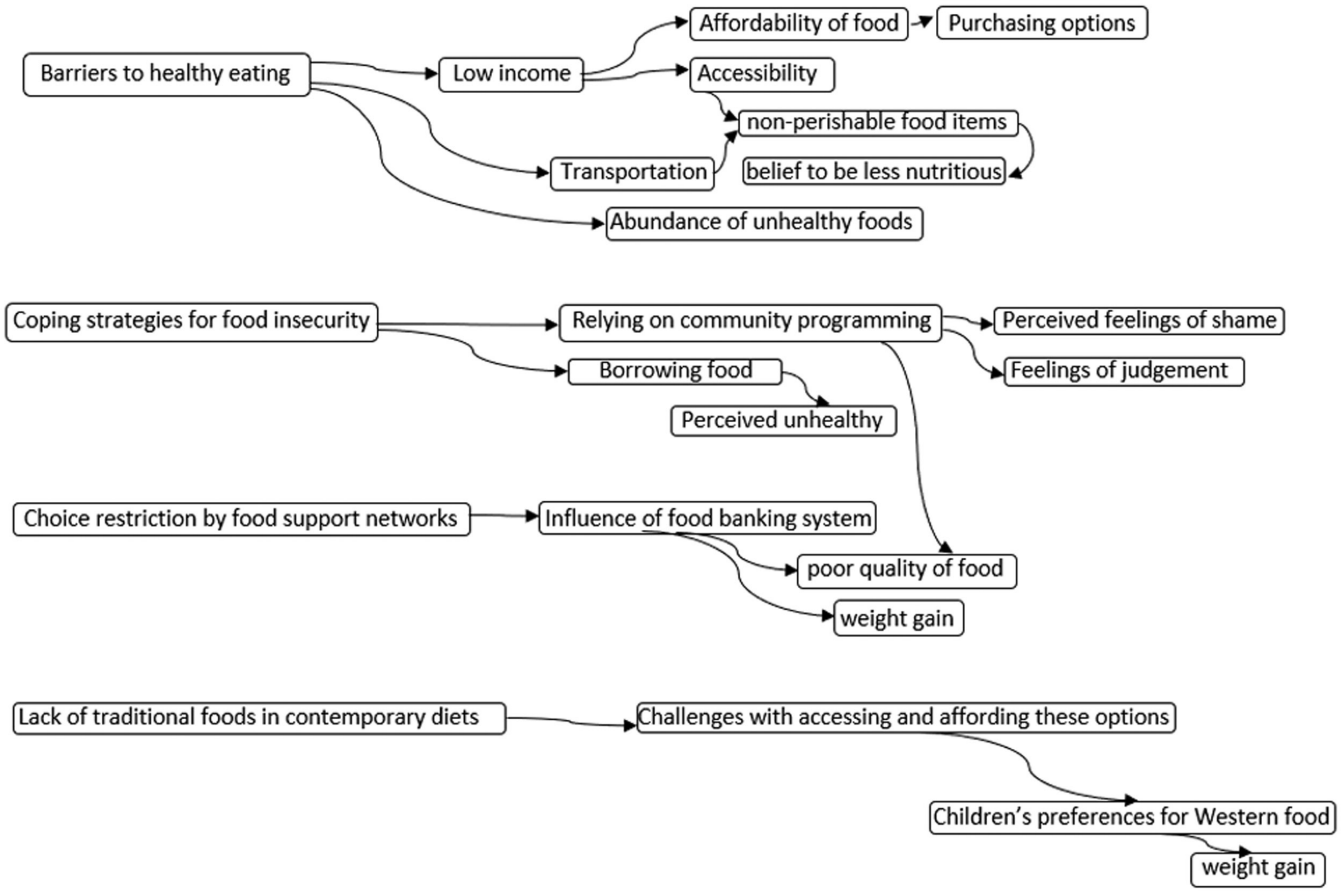
Relations between descriptive themes

Two analytical themes were developed from 19 study findings extracted from included studies. The study findings and relations between descriptive themes are presented in Table S6 and Figure 3. The synthesized analytical findings are as below.

1. A reliance on energy‐dense, nutrient‐poor foods due to their affordability, accessibility, and extended shelf life must be acknowledged. Policy efforts are needed to focus on affordability and availability of neighborhood fresh produce as well as to consider the importance of the food environment in mediating the relationship between food poverty/insecurity and BMI among low‐income individuals.
2. Food banks and other food support networks, used as a coping strategy for food insecurity, have the potential to affect their users' health and body weight. Therefore, increasing the nutritional quality of food provided by them is essential.

#### 3.3.3 Mixed‐methods aggregation of qualitative and quantitative synthesized findings

Mixed‐methods syntheses were conducted to answer the following questions: “Are the results from each synthesis supportive or contradictory?”, “Do the qualitative data support to explain variations in the direction and size of correlations within the included quantitative studies?”. To facilitate the final aggregation of the individual syntheses, a convergent segregated approach was applied (Stern, Lizarondo, Carrier, et al., 2020). The synthesized results from the qualitative components were combined with textual descriptions translated from quantitative findings (Table S7). The mixed‐method synthesis: To reduce the prevalence of overweight/obesity, holistic interventional approaches are required to be implemented to remediate both food insecurity and unhealthy individuals' dietary behaviors that are influenced by different types of food environments. These efforts should emphasize affordability and availability of neighborhood fresh produce as well as to consider other components of food environments such as unhealthy obesogenic food environments in mediating the relationships between food insecurity and overweight/obesity, especially among low‐income families. It is essential that the nutritional quality of food provided by nutrition assistance programs is improved.

## 4 DISCUSSION

The results of our meta‐analysis (*n* = 36,113) showed an overall small, but statistically significant, association between food insecurity and obesity. These results demonstrated that food insecurity increased risk of obesity among adults and children. Therefore, individuals experiencing food insecurity were more likely to be affected by obesity. These findings are important given the context of the “cost of living crisis” and rising health inequalities (Limb, 2022).

There were also positive associations between different types of food environments and overweight and obesity. A study of female participants in SNAP program demonstrated that availability of unhealthy foods at home was significantly associated with BMI (Sanjeevi et al., 2018). This study indicated that the relationship between food insecurity and obesity was partially mediated by home food environment. A large study revealed that women with severe food insecurity had lower odds of obesity if they were usually or always able to afford fresh fruits and vegetables in their neighborhood (Ro & Osborn, 2018). Therefore, neighborhood affordability of fresh produce was accounted as the driving factor that reduced the statistical association between food insecurity and overweight/obesity. Another large study of children aged 2–8 years demonstrated that the average child and adolescents in SNAP household spent a higher percentage of their budget on sugary beverages than on non‐SNAP households (Gorski Findling et al., 2018). In another study of adult residents of low‐income neighborhoods, those who obtained food from charitable sources such as food bank or soup kitchens had significantly higher BMI than those who shopped at convenience stores and those who ate fast‐foods in the month before the survey (Webb et al., 2008).

In the absence of data on the direct associations between food environment exposure and BMI outcome, the links between food insecurity (as outcome) and different types of food environments (as exposure) were also examined. This was performed to better understand the mechanisms behind the association between food environments and weight status as the links between food insecurity and overweight/obesity was established through our meta‐analysis. For example, a study of 4589 middle and high school students did not provide the association between BMI and home food environments directly (Widome et al., 2009). However, it showed that youths with food insecurity had several eating‐related risk factors for overweight/obesity. It demonstrated that these youths, who had the greater percentage of obesity, reported eating more fast‐food than did those who experienced food security. This group also had less both healthy and unhealthy food available in their home. They were also less likely to eat family meals than their counterparts experiencing food security. The study suggested that this might be because of limited or irregular food availability, leading to less instilling a regular family meal routine (Widome et al., 2009). In another population‐based study, the food environment in households living with food insecurity was poorer than in households living in food security (Bruening et al., 2012). Large differences in perceived access to FV were available between parents experiencing food security and parents who experienced food insecurity. Parents who experienced food insecurity reported that the quality and variety of available FV were poor. More importantly, this group of parents perceived that fruits were too expensive to purchase compared to parents experiencing food security (Bruening et al., 2012). Another study of parents and caregivers of kindergarten‐aged children found food security status influenced the parents' experience of obstacles to having healthful food in their home (Bauer et al., 2012). Parents with food insecurity were most likely to report that there was little variety of fruit and vegetables in poor condition where they buy groceries. It also found youths from families who experienced very low food security reported eating more than twice ready‐made food or hot food from a convenience store or compared to children whose families experienced food security. In comparison to children living in food security, youths living with food insecurity also consumed fried chicken and pizza more often (Bauer et al., 2012). The findings from qualitative studies (*n* = 409 participants) regarding a reliance on energy‐dense, nutrient‐poor foods due to their affordability and accessibility aligned with quantitative studies. A study of children's lived experience found that participants mentioned healthy food is more expensive and cost was a barrier to purchasing fresh fruit (Genuis et al., 2015). They also raised the issue that accessibility and transportation play a key role as to reach the closest grocery store that sells a full range of healthy market choices, and that a vehicle is required. Also, findings from qualitative and quantitative studies regarding the potential links between increased body weight and participation in food assistance programs such as food banks, used as a coping strategy for food insecurity, were supportive. A qualitative study of food bank users revealed that although relying on food bank parcels meant that they could afford to pay bills, however, it also meant sacrificing fresh food that exacerbated their weight gain (Thompson et al., 2018).

In the present study, our findings from the aggregation of qualitative and quantitative analyses recommend that holistic approaches (including policy) are required to remediate food insecurity and unhealthy individuals' dietary behaviors that are influenced by different types of food environments in order to reduce the prevalence of overweight/obesity.

### 4.1 Strengths and limitations

This review is particularly comprehensive by the inclusion of both quantitative and qualitative studies. However, the study is not without its limitations. The focus on the impact of both food insecurity status and the food environment on high BMI meant that articles that only measured one of these factors were excluded from this review. This could potentially limit the inclusion of relevant evidence. For example, those studies that considered the important role of smartphone technology but not the role of food insecurity on obesity risk were excluded, although food environments particularly expand into online settings that shape consumers' food choices (Vadiveloo et al., 2021). Most included quantitative studies were cross‐sectional in their designs. Thus, it was not possible to identify causality or direction among key variables (Ro & Osborn, 2018). Moreover, it was not possible to assess whether food insecurity status and/or the food environment variables temporarily caused different behaviors and perceptions or if all these factors shared a common cause (Widome et al., 2009). Therefore, further longitudinal studies are warranted to acknowledge the possible associations between these variables. For those studies that used self‐reported measures to evaluate anthropometric indices, they are subject to misclassification of subjects (Ro & Osborn, 2018). For example, if overweight/obesity were underestimated in those studies, these might make a more cautious interpretation of our results. Regarding food environment exposures, some studies used personal perceptions of neighborhoods. For instance, in a cross‐sectional study of 5957 individuals, neighborhood measures were personal perceptions of neighborhoods. As such, this may not represent objective neighborhood characteristics such as food (Ro & Osborn, 2018). Small sample size of qualitative studies (*n* = 11) could be also considered a limitation. These studies took place in specific context and communities which might limit transferability of findings from such small sample size. However, they provide some insights into the lived experience of individuals suffering from overweight or obesity and food insecurity. Finally, it is difficult to make comparisons between studies in relation to measurement tools used, as these differed from study to study. Measures of food insecurity used by included studies varied in their validity and focus on capturing different elements of food security status. For instance, although the USDA Adult Food Security Survey Module is a validated measure of food insecurity, it focuses on food adequacy, failing to capture other elements of food security status such as preferences, safety, and nutrition (Yau et al., 2020).

### 4.2 Implications for policy and practice

This systematic review highlights that obesogenic food environments and food insecurity significantly contribute to obesity. This supports the evidence concerning reliance on cheap energy‐dense foods in favor of nutrient‐dense foods such as fruits and vegetables. For instance, this review indicates that those living with food insecurity have higher fruit and sugary beverage intakes compared to those living in food security. Since these beverages are cheaper than the equivalent whole fruits, this might be preferred by these individuals under economic constraint (Yau et al., 2020). Since this review indicated that BMI is significantly higher for those who acquire their foods form charitable sources such as food banks, implementing policies and efforts to improve the nutritional quality of food parcels is essential to help food bank users to meet their individual dietary needs. A recent mixed‐method systematic review has explored the nutritional quality of food parcels provided by food banks and the effectiveness of food banks at reducing food insecurity in developed countries (Oldroyd et al., 2022). The results of this study revealed that pre‐packaged food parcels provided by food banks were inconsistent at meeting nutritional requirements of their users and often failed to meet individual needs, including cultural and health preferences. Use of food banks improved food security and dietary quality of users, allowing otherwise unachievable access to food. Nevertheless, food insecurity remained, and was explained by limited food variety, quality, and choice (Oldroyd et al., 2022).These mixed‐method findings encourage interventions to ensure consistent, adequate nutrition, and improved nutritional quality of food parcels at food banks to meet nutritional needs of those requiring food banks.

These findings emphasize the importance of structural and policy changes to the food and economic environment. There need to be societal changes to reduce inequalities to facilitate national and international goals of reducing overweight and/or obesity. This also provides scope for halting rising trends in food insecurity as well as eradicating food insecurity. A suggested approach to tackling such issues might be to address the high and rising cost of food, especially healthy foods (Yau et al., 2020) particularly within the context of the global cost of living crisis. It is reported that in high‐income countries like the UK, even people who work full‐time on the National Living Wage cannot necessarily achieve the Minimum Income Standard (i.e., the income needed to reach a minimum socially acceptable standard of living; Yau et al., 2020). Therefore, combined with the rising economic crises related to recent COVID‐19 pandemic and world events, addressing wage‐related policies to ensure sufficient income for adequate standards of living is critical to address health inequalities.

### 4.3 Implications for research

Further longitudinal studies investigating the impact of obesogenic food environments and food insecurity on obesity among general populations, rather than minority‐specific, and in countries beyond the USA, will strengthen the evidence base. Since this review indicated that BMI is significantly higher for those who acquire their foods from charitable sources such as food banks, further updated reviews for high‐income countries investigating the nutritional quality of food parcels and whether using foodbanks reduces the food insecurity and improves their users' diets will strengthen the evidence base.

## 5 CONCLUSIONS AND IMPLICATIONS OF THIS REVIEW

Drawing on evidence from research across high‐income countries, the present systematic review and meta‐analysis showed that food insecurity and some types of food environments are a risk factor for obesity. Wide‐reaching approaches (including policy changes) are required to address overweight/obesity among individuals experiencing food insecurity, especially among those whose food choices are influenced by unhealthy food environments. Our results suggest that these efforts should focus on affordability and availability of neighborhood fresh produce as well as to consider other components of food environments such as unhealthy obesogenic food environments in mediating the relationships between food insecurity and overweight/obesity, especially among low‐income families. It is also essential that the nutritional quality of food offered by nutrition assistance programs is improved.

## CONFLICT OF INTEREST

No conflicts of interest.

## ETHICAL STATEMENT

This review used only published sources of data. Ethical review by a Research Ethics Committee was not required.

## Supporting information


Tables S1–S7

## Data Availability

The data that support the findings of this study are available from the corresponding author upon reasonable request.
