# Supplementary material for: A mixed‐method systematic review and meta‐analysis of the influences of food environments and food insecurity on obesity in high‐income countries
Source: Food Sci Nutr. 2022 Aug 5;10(11):3689–723. doi: 10.1002/fsn3.2969 (PMC9632201; doi:10.1002/fsn3.2969)
Supplement: Supplementary file 1 — Tables S1–S7 [file FSN3-10-3689-s001.docx]

**Table S1** **– Key words identified in development of search strategy**

| **Population** | | **Exposure of Interest (Independent variable)** | | | **Outcome (Dependent variable)** |
| --- | --- | --- | --- | --- | --- |
| Adult | Children | Geography | Food environment | Food insecurity | Obesity |
| Men | Teenager | Geographic or geospatial | Foodscape* | Food poverty | Overweight |
| Women | Adolescent | geolocation | Food desert* | Food hardship | Body fatness |
| Male | Kid | Geographic information systems or GIS | Food landscape* | Food assistance | Excessive weight |
| Female | Toddler | Geographic mapping | Food supply | Food deprivation | Extra weight |
| Elder | Infant | Spatial analysis | Food access | Food aid* | Above normal weight |
| People | Minor | Satellite imagery | Nutrition environment | Hunger | Weight gain or increase |
|  | Juvenile | Remote sensing technology | Fast-food retail store | Food banks | Body mass index or BMI |
|  | Paediatric | Environmental monitoring | Fast-food restaurants | Food pantries | Fat |
|  |  | Neighbourhood | Convenience store | Food inequalities |  |
|  |  | Situation | Supermarket | Food security |  |
|  |  | Setting | Grocery store |  |  |
|  |  | Reign | Chain store |  |  |
|  |  | District | Food outlet |  |  |
|  |  | Rural areas | Food availability |  |  |
|  |  | Urban areas | Food adequacy |  |  |

**Table S2** **– Full search example; PubMed search**

| **Search terms** | **Add to builder** | **Hits** | **EndNote/details** | **Hits 28/7/2020** | **Hits 24/8/2021** |
| --- | --- | --- | --- | --- | --- |
| Search ((((((((((((((((((geography) OR geographic) OR geospatial) OR geolocation) OR geographic information systems) OR GIS) OR geographic mapping) OR spatial analysis) OR satellite imagery) OR remote sensing technology) OR environmental monitoring) OR neighbourhood) OR urban areas) OR rural areas) OR situation) OR setting) OR region) OR district) OR area | OR | 6017965 |  |  |  |
| Search ((((((((((food insecurity) OR food poverty) OR food hardship) OR food assistance) OR food deprivation) OR food aid*) OR hunger) OR food banks) OR food pantr*) OR food inequalities) OR food security | OR | 83006 |  |  |  |
| Search (((((((((((((((food environment) OR foodscape) OR food deserts) OR food desert) OR food landscape) OR food supply) OR food access) OR nutrition environment) OR fast food retail store) OR fast food restaurant) OR convenience store) OR supermarket) OR grocery store) OR chain store) OR food availability) OR food adequacy | OR | 223549 |  |  |  |
| Search ((((((((((obesity) OR overweight) OR excessive weight) OR extra weight) OR above normal weight) OR weight gain) OR weight increase) OR body mass index) OR BMI) OR body fatness) OR body fatness fat | OR | 638286 |  |  |  |
| **Search ((((((((((((((((((((((geography) OR geographic) OR geospatial) OR geolocation) OR geographic information systems) OR GIS) OR geographic mapping) OR spatial analysis) OR satellite imagery) OR remote sensing technology) OR environmental monitoring) OR neighbourhood) OR urban areas) OR rural areas) OR situation) OR setting) OR region) OR district) OR area)) AND (((((((((((food insecurity) OR food poverty) OR food hardship) OR food assistance) OR food deprivation) OR food aid*) OR hunger) OR food banks) OR food pantr*) OR food inequalities) OR food security)) AND ((((((((((((((((food environment) OR foodscape) OR food deserts) OR food desert) OR food landscape) OR food supply) OR food access) OR nutrition environment) OR fast food retail store) OR fast food restaurant) OR convenience store) OR supermarket) OR grocery store) OR chain store) OR food availability) OR food adequacy)) AND (((((((((((obesity) OR overweight) OR excessive weight) OR extra weight) OR above normal weight) OR weight gain) OR weight increase) OR body mass index) OR BMI) OR body fatness) OR body fatness fat)** | **AND** | 2664 | Downloaded to EndNote 24/1/2019 | 431 | 396 |

**Table S3** **– Table for quality assurance assessment for cross sectional studies**

| **Study** | **1. Were the criteria for inclusion in the sample clearly defined?** | **2. Were the study subjects and setting described in detail?** | **3. Was the exposure measured in a valid and reliable way?** | **4. Were objective, standard criteria used for measurement of the condition?** | **5.Were confounding factors identified?** | **6. Were strategies to deal with confounding factors stated?** | **7. Were the outcomes measured in a valid and reliable way?** | **8. Was appropriate statistical analysis used?** | **Total/8** |
| --- | --- | --- | --- | --- | --- | --- | --- | --- | --- |
| **Ro A and Osborn B., 2018** |  |  |  |  |  |  |  |  | **8** |
| **Bruening et al., 2012** |  |  |  |  |  |  |  |  | **8** |
| **McCurdy et al., 2015** |  |  |  |  |  |  |  |  | **8** |
| **Sanjeevi et al., 2018** |  |  |  |  |  |  |  |  | **7** |
| **Vedovato et al., 2016** |  |  |  |  |  |  |  |  | **8** |
| **Robaina and Martin., 2013** |  |  |  |  |  |  |  |  | **7** |
| **Web et al., 2008** |  |  |  |  |  |  |  |  | **8** |
| **Bauer et al., 2012** |  |  |  |  |  |  |  |  | **6** |
| **Leung and Villamor., 2011** |  |  |  |  |  |  |  |  | **8** |
| **Kaiser et al., 2019** |  |  |  |  |  |  |  |  | **8** |
| **Nguyen et al., 2015** |  |  |  |  |  |  |  |  | **7** |
| **Dharod et al., 2013** |  |  |  |  |  |  |  |  | **8** |
| **Mercile et al., 2012** |  |  |  |  |  |  |  |  | **6** |
| **Gorski et al., 2018** |  |  |  |  |  |  |  |  | **8** |
| **Widome et al., 2009** |  |  |  |  |  |  |  |  | **8** |
| **Poulsen et al., 2019** |  |  |  |  |  |  |  |  | **8** |
| **Matheson et al., 2002** |  |  |  |  |  |  |  |  | **6** |
| **Kral et al., 2017** |  |  |  |  |  |  |  |  | **8** |
| **Watt et al., 2013** |  |  |  |  |  |  |  |  | **8** |
| **Smith and Richards., 2008** |  |  |  |  |  |  |  |  | **6** |
| **Yau et al., 2020** |  |  |  |  |  |  |  |  | **8** |
| **Keenan et al., 2021** |  |  |  |  |  |  |  |  | **8** |
| **Shinwell et al., 2021** |  |  |  |  |  |  |  |  | **8** |
| **Niu., et al., 2021** |  |  |  |  |  |  |  |  | **8** |
| **Huelskamp et al., 2021** |  |  |  |  |  |  |  |  | **6** |
| **Walch, A, and Holland, K. 2021** |  |  |  |  |  |  |  |  | **8** |
| **Rodriguez et al., 2021** |  |  |  |  |  |  |  |  | **8** |
| **Domingo et al., 2021** |  |  |  |  |  |  |  |  | **8** |
| **Santarossa et al., 2021** |  |  |  |  |  |  |  |  | **8** |
| **Wirth et al., 2020** |  |  |  |  |  |  |  |  | **8** |
| **van der Velde., 2020** |  |  |  |  |  |  |  |  | **8** |
| **Vadiveloo et al., 2020** |  |  |  |  |  |  |  |  | **8** |
| **Wilcox et al., 2020** |  |  |  |  |  |  |  |  | **8** |
| **Nettle, D., and Bateson M. 2019** |  |  |  |  |  |  |  |  | **8** |

**Table S4** **– Table for quality assurance assessment for cohort studies**

| **Study** | **1. Were two groups similar and recruited from the same population?** | **2. Were exposures measured similarly to assign people to both exposed and unexposed groups?** | **3. Was the exposure measured in a valid and reliable way?** | **4. Were confounding factors identified?** | **5. Were strategies to deal with confounding factors stated?** | **6. Were groups/participants free of the outcome at the start of the study (or at the moment of exposure)?** | **7. Were the outcome measured in a valid and reliable way?** | **8. Was the follow up time reported and sufficient to be long enough for outcomes to occur?** | **9. Was follow up complete, and if not, were the reasons to loss to follow up described and explored?** | **10. Were strategies to address incomplete follow up utilized?** | **11. Was appropriate statistical analysis used?** | **Total/11** |
| --- | --- | --- | --- | --- | --- | --- | --- | --- | --- | --- | --- | --- |
| **Benjamin-Neelon et al., 2020** |  |  |  |  |  |  |  |  |  |  |  | **11** |

**Table S5** **– Table for quality assurance assessment for qualitative studies**

| **Study** | **1. Is there congruity between the stated philosophical perspective and the research methodology?** | **2. Is there congruity between the research methodology and the research question or objectives?** | **3. Is there congruity between the research methodology and the methods used to collect data?** | **4. Is there congruity between the research methodology and the representation and analysis of data?** | **5. Is there congruity between the research methodology and the interpretation of results?** | **6. Is there a statement locating the researcher culturally or theoretically?** | **7. Is the influence of the researcher on the research, and vice-versa, addressed?** | **8. Are participants, and their voices, adequately represented?** | **9. Is the research ethical according to current criteria or, for recent studies, and is there evidence of ethical approval by an appropriate body?** | **10. Do the conclusions drawn in the research report flow from the analysis, or interpretation, of the data?** | **Total/10** |
| --- | --- | --- | --- | --- | --- | --- | --- | --- | --- | --- | --- |
| **Bhawra et al., 2015** |  |  |  |  |  |  |  |  |  |  | **10** |
| **Franzen and Smith., 2009** |  |  |  |  |  |  |  |  |  |  | **10** |
| **Genuis et al., 2015** |  |  |  |  |  |  |  |  |  |  | **10** |
| **Kerpan et al., 2015** |  |  |  |  |  |  |  |  |  |  | **10** |
| **Thompson et al., 2018** |  |  |  |  |  |  |  |  |  |  | **10** |
| **Holston et al., 2021** |  |  |  |  |  |  |  |  |  |  | **7** |
| **Cooksey Stowers et al., 2020** |  |  |  |  |  |  |  |  |  |  | **9** |
| **Ong et al., 2021** |  |  |  |  |  |  |  |  |  |  | **9** |
| **Byker Shanks., et al., 2020** |  |  |  |  |  |  |  |  |  |  | **9** |
| **Gosliner, W. and Shah, HA. 2020** |  |  |  |  |  |  |  |  |  |  | **9** |
| **Jennings et al., 2020** |  |  |  |  |  |  |  |  |  |  | **9** |

**Table S6** **– Qualitative Findings with illustrations**

Bhawra et al. 2015

| Finding | Illustration |
| --- | --- |
| Low income as a main determinant (affordability): there are connections between cost of food and an impact on provision | “So, you know, the fattier foods are the lowest price and they go a lot further. So, you’re going to see obesity in that stereotypical low-income/one income family. And if you take a two-income family, yes, you know what, there’s more money coming in. So, yes, they can get the fresh fruits, they can get the fresh vegetables, they can buy the milk, they can, you know, they don’t have to live on Kraft Dinner and soup”. p5 |
| Accessibility and transportation play a key role in prioritizing the shelf-life above healthy choices | “I think it’s because sometimes when you buy fruits and vegetables, they tend to, like the shelf-life is not as long as the other foods. We just recently moved to the reserve [adjacent to the city] and you need a vehicle to get to town and buy those foods like every so often […], and a lot of people that live on the reserve, they go grocery shopping maybe once or twice a month, so they’re not able to continuously get fruits and vegetables”. p6 |
| Affordability and accessibility to traditional foods: some foods available but unobtainable due to cost | “You can get it, but it’s expensive. And not all of the traditional foods are easy to get, like wild game, is not easy to get”. p6 |
| Reliance upon community food initiatives as coping strategies for food insecurity | “Participants identified numerous barriers to programming that either hindered their participation or the programs’ effectiveness. Caregivers specifically spoke about the food box programs and food banks within their communities. They felt that these programs often had food of poor quality that was either near to or past its expiration date. In addition to subpar food options, many caregivers recalled feelings of shame associated with using food banks. The feelings of judgment exacerbated the stigma caregivers already felt were associated with receiving food charity”. p7 |

Franzen and Smith 2009

| Finding | Illustration |
| --- | --- |
| Affordability and lack of resources (time) results in higher use of convenience foods. | Majority of participants reported consuming processed or convenience foods due to cost of fresh foods, busy lifestyles, and cooking time needed for traditional food. As one mentioned, ‘‘when you get off work you’re so lazy you just don’t feel like cooking all that food and I think Hmong food takes a lot of preparation, so we’ll just throw in like a pizza or we’ll make spaghetti, something really fast or ramen noodles (B-US, F)’’. p178 |
| Increased access to energy dense foods (availability) | “Nutrition transitions experienced by the Hmong included decreased consumption of rice in younger generations, altered food preparation methods, increased consumption of convenience/processed foods, the introduction of snacking, and food assistance programs introducing foods outside cultural norms to younger generations” p178 |
| Traditional eating patterns: affordability and perception that fruits and vegetables are not important being barrier to consumption. | ‘‘. . .sometimes it was too expensive and they didn’t have enough [money] to buy [it] or sometime they think that fruit is not important like the other food [vegetables, rice] to support a family (B-TL1, M).’’ p177 |
| Food assistance programmes seen as being responsible for introducing immigrants to unhealthy foods, encouraging consumption of high fat/high sugar foods. | “I realized that I was introduced to American food through WIC and the FSP because we were forced to eat it (B-US, F)”. p178 |

Genuis et al. 2015

| Finding | Illustration |
| --- | --- |
| Barriers to healthy eating: affordability of fresh fruit (healthy food) | Cost was a barrier to purchasing fresh fruit as mentioned by two participants. One stated that he did not consume berries very often as ‘they cost too much’; the second participant mentioned ‘Sometimes [my mother] doesn’t have that much money, so she can’t afford [fruit]’. The study concluded that healthy food is more expensive. p604 |
| Accessibility and transportation play a key role in provision of healthy foods. | “A vehicle is required to drive off the reserve to reach the closest grocery store that sells a full range of healthy market choices on a year-round basis” p604 |
| Dominance of photos depicting packaged, quick-preparation foods – high sugar cereal items | “just over 50% of the photos identified as breakfast were boxed breakfast cereals – the majority being those which are high in sugar” p604 |

Kerpan et al. 2015

| Finding | Illustration |
| --- | --- |
| Competing priorities: borrowing food (as a coping strategy for food insecurity) perceived unhealthy. | “One month we spend all our money on rent and we just had to bum food off my stepmom’s brother and all he had was junk food,” said one participant. |
| Barriers to healthy eating: affordability of fresh fruits and vegetables (healthy food) | “I want vegetables and fruits and stuff like that but I can’t fit them in the budget,” said a participant who lived on her own. |
| Choice restriction by food support networks | Participants often used food banks. “It’s hard for some people in the city to eat healthy because in those places that give out food you don’t really get a choice in what you get, you get what they give.” Participants frequently stated the food from the food bank was inadequate. “Sometimes you don’t get very good stuff” and “Its ugly food and expired.” p397 |
| Logistically difficult to obtain the healthy food, access transport due to location and waiting times/length of travel time, all seen as barriers to healthy eating. | Participants said that taking the bus “takes a long time,” and they often “had to wait outside  for the bus and then pack groceries on the bus.” p397 |
| Food swamps make it difficult to choose healthy options. | “I live by A&W, McDonalds, KFC, and three other restaurants like that. So it’s pretty hard [to eat healthy], lots of fatty foods.” Another respondent mentioned that the problem is not only related to fast-food restaurants: “I live by a store. That’s where I get chips and pop. It’s a corner store, like a confectionary.” A young man acknowledged that there were some healthy items at the corner stores near his house, but making a healthy choice was a challenge: “You see a whole shelf of chips, and then beside it there is a little one with bread. That does make an impact, even if you do not realize it. Because then you’re looking at the chips even though you need bread”. p397 |

Thompson et al. 2018

| Finding | Illustration |
| --- | --- |
| Competing priorities: bills need to be paid but sacrificing fresh food. | "Linda, a working single mother, explained that relying on food bank parcels meant that she could afford to pay the rent. But it also meant going without fresh food. It does have an impact on health, because you don't get anything fresh, I mean that's just the nature of it, everything's packaged in packets and jars and tins, and obviously it's people that have donated things…" p99 |
| Food poverty exacerbates weigh-gain. | “Experiencing food poverty can create new health and social problems and worsen existing ones. Participants experiencing food poverty explained that issues such as stress, depression and weight-gain were made worse by these experiences and more difficult to manage”. p100 |
| Food banks unable to meet the needs of users with special medial problems. | “she eventually requested a referral to a food bank to save money for her prescription medications. However, as has been described above, constructing specialised and restricted diets (like that following bariatric surgery) from donated food is very challenging. As Bill (a GP in a deprived London borough) explains below:  She was very you know upset … had been going to the food bank to get sort of a limited amount of food, couldn't get any of her prescriptions …”. p100 |

Holston et al., 2021

| Finding | Illustration |
| --- | --- |
| Barriers to healthy eating: affordability of healthy food | “To feed somebody healthy . . . it costs way more. A lot of people are pretty much looking at food that’s filling. You know what I’m saying? So you don’t have to eat as much. Like rices and starches and stuff like that to make sure food can go further, I guess. But healthy food, it really costs”. p8 |
| Accessibility, affordability, and transportation play a key role in provision of healthy and better-quality foods. | “I get food stamps. I’m not ashamed of that. I’m proud of that. That’s a blessing . . . But the thing of it is, I be trying to stretch it, you know. That’s what I be trying to do is stretch it. If I go to [local store], I ain’t get nothing on that. And the meats are so high there . . . I find those meats and things cheaper when I go [out of parish] than I do at these stores around here”. P5 |
| Price as the most important deciding factor for almost all participants. | “We compare these prices. We get the sale paper and we compare the price. And we also go for the cheapest”. Participants were aware of small differences in price: “it could be a penny difference, I’m still going there (laughs). Things add up”. P5 |

Cooksey Stowers et al., 2020

| Finding | Illustration |
| --- | --- |
| Access to unhealthy food from donors (Structural inequity themes) | “I think the way the food bank system was designed, it was designed to take any surplus food that was available and distribute it. . . this long held fear of losing donors—and by donors I mean corporate food and beverage donors. . . who are donating these large quantities, donations of food. You know, the good, the bad and the ugly”. P5 |
| Food banks unable to meet the needs of users with special medial problems. | “There’s a lack there [in the food bank system] of getting certain very low sodium, very low fat, high fibre—the kind of things that the folks who are dealing with diabetes and heart disease and other health issues. When their doctor says, take with food, change your diet, maybe you have to be gluten-free now because we’re finding celiac disease, how can food banks respond and get the food that’s needed to not only serve a vulnerable population just in the way of food insecurity, but probably also a population that has some fairly specific food needs”. P5 |
| Privilege of access to information (social theme) | “Those who are educated, they may not necessarily be wealthy, but those who are educated about the need to eat more healthfully—and they have the know-how on how to cook those items, those more wholesome items, too”. P |

Ong et al., 2021

| Finding | Illustration |
| --- | --- |
| Barriers to healthy eating: affordability of healthy food (sacrificing nutritional quality to obtain free food or food at a discount because of inadequate income to support healthy diets) | “You know what happened if you rely on food bank … it's not balanced diet … Processed food actually puts you more weight … and I decided to eat that instead of giving to my child. I use the money to buy fruit and veggies for him … People with low income … they're going to get sick, and they don't have the money for the medication … it's gonna be a cycle of problem in there. — Josie (Josie, Latin American, female in her 40s, experiencing moderate food insecurity)”. P6 |
| Barriers to healthy eating: affordability of healthy food | “In the summer I go up to the farmers' market to get stuff because it's really fresh, but the winter unfortunately I go to [corporate grocer] because it's really cheap — Iván (white male, age 51–64, experiencing severe food insecurity)”. P4 |
| Barriers to healthy eating: affordability of healthy food | “I’m on the autism spectrum and the specialist in that area recommended that I eliminate pasta, bread, a whole bunch of things … If I were to get what she was suggesting I get, it would quadruple my budget and require so much work for me. — John (white male, senior citizen, experiencing moderate food insecurity)”. P6 |

Byker Shanks et al., 2020

| Finding | Illustration |
| --- | --- |
| Transportation plays a key role as barriers to food access | “They don’t have a car or, if they do have a car, they don’t have a truck to go into the mountains to get fresh berries and things like that”. P |
| Lack of food availability | “Nothings organic, everything’s non-organic, vegetables and fruit, they don’t have fresh sometimes, it’s like kind a rotten and then they don’t really have meat too much there and if they do its all processed meats, so hot dogs and lunch meats [ …] and they have a lot of pop, they have a ridiculous amount of pop”. P |

Gosliner, W. and Shah, HA. 2020

| Finding | Illustration |
| --- | --- |
| Local food environment not perceived as supportive of healthy eating | “Hamburgers and everything is like US$1, but salads are like US$5. They make it hard for people to want to eat healthy when it costs so much. The one that’s healthy should be cheaper than a hamburger.”. P411  “…because when you go to the grocery store, the first thing you see are the chips, cookies, and sodas on sale”. P411 |
| Barriers to healthy eating: affordability of healthy food; (cost as a barrier to provide healthy foods) | “And we don’t have that type of money to be buying fresh vegetables, fresh fruits—we don’t have it like that. [Another participant] Understand that fresh vegetables, fresh fruits really cost a lot of money…”. P411  “At the end of the month, you ain’t got no money. You’re struggling to have food up until the last day… so sometimes it’s hard to eat that way [referring to the MyPlate recommendations].”. P411 |
| Barriers to healthy eating: affordability of healthy food; (inadequate financial resources, low wages, and high costs of living) | “You feel it, too, but it’s a lot of pressure when you’re trying to run your house, you’re trying to feed people, and you’ve just got all your daily stresses just doing this to you. So it’s very, very hard. And then the wages that they pay a lot of us – it’s not helping. US$10.50 an hour?… There’s no break… You’re surviving, that’s it. There’s no comfort.”. P411  “The jobs out here aren’t paying enough for these people to live, let alone pay rent. That’s why a lot of people are on Section 8. Because the wages don’t allow us to pay rent. Everybody can’t have US$14.00, 15.00,$16.00 an hour job”. P411 |

Jennings et al., 2020

| Finding | Illustration |
| --- | --- |
| Challenges with affordability of fruits/vegetables. | “Several reported that they could get “addicted” to healthy food, as they enjoyed the flavor and idea of healthiness; however, after observing the prices at the market, they worried about affordability”. P898 |
| Poverty as root cause of food insecurity | “The participants noted that their early life has been and still is affected by poverty (e.g., not having enough money to purchase food and not knowing what they would eat for the next meal) and homelessness”. P899 |
| Food swamps (unhealthy food easily accessible), and affordability as barrier to healthy food options. | Vending machines: “Quick and cheap and has tasty food. But it is unhealthy because it’s too sugary and has lots of fat”, Supplementary Material |
| Accessibility, transportation, as barrier to healthy food options. | “Not having transportation to food can make you starve so when you finally have the food you overeat because you are insecure about having food tomorrow”. Supplementary Material  “We don’t have a car now and it’s too cold in the winter. My mom tells me it’s unsafe to walk to the grocery store”. Supplementary Material |
| Food insecurity and obesity | “When you feel you’re gonna lose the food and you keep eating it, it makes you gain weight which makes you increase your weight and your body will need more calories when you’re fat so you can stay alive, but if you’re fat you can get diseases”. Supplementary Material |

**Table S7** **–** **Mixed methods aggregation of qualitative and quantitative synthesised findings**

| **Qualitative synthesised findings** | **Textual description of quantitative findings** | **Mixed method synthesis** |
| --- | --- | --- |
| **Synthesised finding 1:** A reliance on energy-dense, nutrient-poor foods due to their affordability, accessibility and extended shelf life must be acknowledged. Policy efforts are needed to focus on affordability and availability of neighbourhood fresh produce as well as considering the food environment in mediating the relationship between food poverty/insecurity and BMI amongst low-income individuals. | Food insecurity increases overweight/obesity and there are links between different types of food environments and with higher BMI. | To reduce the prevalence of overweight/obesity, holistic interventional approaches are required to be implemented to remediate both food insecurity and unhealthy individuals’ dietary behaviours that are influenced by different types of food environments. These efforts should focus on affordability and availability of neighbourhood fresh produce as well as considering other components of food environments such as unhealthy obesogenic food environments in mediating the relationships between food insecurity and overweight/obesity, especially among low-income families. |
| **Synthesised finding 2:** Food banks and other food support networks, used as a coping strategy for food insecurity, have the potential to influence users’ health and body weight. So, increasing the nutritional quality of food provided by them is essential. | Participation in different types of food assistance programmes was associated with increased risk of overweight/obesity. | It is essential that the nutritional quality of food provided by nutrition assistance programmes to be improved. |
